# Comparative analysis of protein-protein interactions in the defense response of rice and wheat

**DOI:** 10.1186/1471-2164-14-166

**Published:** 2013-03-12

**Authors:** Dario Cantu, Baoju Yang, Randy Ruan, Kun Li, Virginia Menzo, Daolin Fu, Mawsheng Chern, Pamela C Ronald, Jorge Dubcovsky

**Affiliations:** 1 Department of Viticulture & Enology, University of California Davis, Davis, CA, USA; 2 Department of Plant Sciences, University of California Davis, Davis, CA, USA; 3 Northwest A&F University, Yangling, Shaanxi, China; 4 Department of Plant Pathology, University of California Davis, Davis, CA, USA; 5 College of Agronomy, Shandong Agricultural University, Tai’an, Shandong, China; 6 CRA, Experimental Institute for Cereal Research of Foggia, Foggia, Italy; 7 Genome Center, University of California Davis, Davis, CA, USA; 8 Howard Hughes Medical Institute, Chevy Chase, MD, USA; 9 Gordon & Betty Moore Foundation, Palo Alto, CA, USA

## Abstract

**Background:**

Despite the importance of wheat as a major staple crop and the negative impact of diseases on its production worldwide, the genetic mechanisms and gene interactions involved in the resistance response in wheat are still poorly understood. The complete sequence of the rice genome has provided an extremely useful parallel road map for genetic and genomics studies in wheat. The recent construction of a defense response interactome in rice has the potential to further enhance the translation of advances in rice to wheat and other grasses. The objective of this study was to determine the degree of conservation in the protein-protein interactions in the rice and wheat defense response interactomes. As entry points we selected proteins that serve as key regulators of the rice defense response: the RAR1/SGT1/HSP90 protein complex, NPR1, XA21, and XB12 (XA21 interacting protein 12).

**Results:**

Using available wheat sequence databases and phylogenetic analyses we identified and cloned the wheat orthologs of these four rice proteins, including recently duplicated paralogs, and their known direct interactors and tested 86 binary protein interactions using yeast-two-hybrid (Y2H) assays. All interactions between wheat proteins were further tested using *in planta* bimolecular fluorescence complementation (BiFC). Eighty three percent of the known rice interactions were confirmed when wheat proteins were tested with rice interactors and 76% were confirmed using wheat protein pairs. All interactions in the RAR1/SGT1/ HSP90, NPR1 and XB12 nodes were confirmed for the identified orthologous wheat proteins, whereas only forty four percent of the interactions were confirmed in the interactome node centered on XA21. We hypothesize that this reduction may be associated with a different sub-functionalization history of the multiple duplications that occurred in this gene family after the divergence of the wheat and rice lineages.

**Conclusions:**

The observed high conservation of interactions between proteins that serve as key regulators of the rice defense response suggests that the existing rice interactome can be used to predict interactions in wheat. Such predictions are less reliable for nodes that have undergone a different history of duplications and sub-functionalization in the two lineages.

## Background

Biotic stresses, caused by highly specialized obligate parasites are among the most damaging diseases of wheat worldwide. While genetic resistance is a cost-effective, safe, and environmentally-sound method of disease control, our current ability to generate durable resistance in wheat is limited by our poor understanding of the genetic mechanisms and gene interactions involved in the wheat resistance response [1].

The interaction between a pathogen and its plant host represents a highly complex and dynamic system. Upon pathogen attack, a complex series of signaling events must occur in order to ensure that the proper cellular responses are activated. Multiple layers of molecular cross-talk between plant and microbial proteins and between plant proteins determine the outcome of the interaction and the intensity of the disease state [2]. The signaling cascades that activate plant defenses are typically initiated following the recognition of pathogen-associated molecular patterns or pathogen effectors by plant receptors or by host detection of pathogen induced modifications [3]. Complex signaling cascades involving kinases and transcription factors (among others) ultimately result in the transcriptional activation of defense mechanisms. The successful biotrophic lifestyle depends upon the ability of the pathogen to avoid or suppress host defenses via perturbations of critical nodes of these defense response pathways [3].

Physical interactions between proteins (interactome) play a critical role in the cascade of events associated with the activation of defense responses to pathogens [4]. For example, protein-protein interactions govern the interaction of host pattern recognition receptors with their ligand and downstream signaling proteins and are also important for effector recognition [5], protein phosphorylation [6] and transcriptional co-factor recruitment [7]. Therefore, a better understanding of the defense response interactome will be helpful for an intelligent manipulation of the resistance pathways, and to select combinations of resistance genes that maximize their durability [1]. Genome-wide interactome maps can be developed using high-throughput experimental techniques, such as yeast two-hybrid system (Y2H; [8]) and mass spectrometry [9]. The systematic mapping on a genome-wide scale of interactions has been undertaken in model plant species whose complete genomic sequences are available, such as *Arabidopsis thaliana*[10,11] and rice [4,8,12]*.* As a result of these efforts high-quality large-scale interaction maps for protein kinase signaling pathways and for the biotic and abiotic stress responses have been developed [4,12]. The rice stress response interactome has already provided a powerful tool for the dissection of the signal transduction pathway mediated by XA21, a pattern recognition receptor [13] that confers resistance to *Xanthomonas oryzae* pv. *oryzae* (*Xoo*; [14]). Genetic analyses of proteins identified through these studies validated their function in the defense response and provided new insights into the signal transduction pathways governing disease resistance [6,15-19].

Unlike rice and Arabidopsis, two diploid species with relatively small genomes (450 Mb and 135 Mb, respectively), commercial wheat species are polyploid (tetraploid for the pasta wheat, *Triticum turgidum*, and hexaploid for the bread wheat, *T. aestivum*) and have large genomes (pasta wheat, 13,000 Mb; bread wheat, 16,000 Mb). The sequence of the non-repetitive portions of the *T. aestivum* genome was only very recently made available ([20], http://urgi.versailles.inra.fr). The complete sequence of the rice genome has provided an extremely useful parallel road map for genetic and genomics studies in wheat. The co-linearity between large blocks of wheat and rice chromosomes allowed the development of high-density maps that have enabled the isolation of genes encoding important wheat agronomic traits [21-27]. Comparative rice and wheat genome analysis have greatly improved gene structure prediction [28] and transcript annotation [29].

Similarly, the recent development of a protein-protein interaction network (interactome) of the rice defense response promises to advance knowledge in wheat and other grasses, which have more limited genomic resources. In order to use the rice stress response interactome as a template for dissection of the protein-protein interaction network controlling defense responses in wheat it is necessary to determine first the degree of conservation of these interactions in these two species. The objective of this work was to evaluate the conservation of these protein-protein interactions. We selected six proteins from the rice stress response interactome that represent key nodes controlling the rice defense response. These include (i) the protein folding chaperon complex RAR1/SGT1/HSP90 [30-33], (ii) the systemic acquired resistance regulator NPR1 (NPR1-homolog1, NH1 [16]), and (iii) the pattern recognition receptor XA21 [14] and (iv) XB12, a shikimate kinase-like protein that interacts with XA21 [4].

The RAR1/SGT1/HSP90 complex plays a critical role in plant immunity [30-33] by mediating the proper folding/stability of many NB-LRR R-proteins [30,34-37]. For example wheat *Lr21*-mediated resistance to leaf rust requires the expression of *RAR1*, *SGT1*, and *HSP90*[38]. NPR1 (also known as NIM1 and SAI1) is a critical regulator in the salicylic acid (SA)-mediated signal transduction pathway that activates the systemic acquired resistance (SAR) responses [39,40]. Arabidopsis *npr1* mutants do not induce PR gene expression and fail to initiate SAR responses upon pathogen challenge or treatment with SA or with the SAR inducer, 2,6-dichloroisonicotinic acid. When up-regulated, the rice NPR1 homolog1 (NH1) confers robust resistance to *Xoo*[16], whereas its down-regulation leads to loss of benzothiadiazole-induced resistance to *Pyricularia oryzae*[41]. The rice *XA21* gene encodes a pattern recognition receptor that confers broad-spectrum resistance to *Xoo*, the causal agent of bacterial blight disease. XA21 consists of an N-terminal leucine rich repeat domain (LRR), a transmembrane domain, a juxtamembrane domain (JM) and a cytosolic C-terminal non-RD (arginine-aspartate) kinase domain [14]. Upon recognition by XA21 of a highly conserved sulfated peptide derived from the Ax21 N-terminal domain [42], XA21-mediated immunity is activated through a signaling cascade involving XA21 JM domain-mediated protein-protein interactions and nuclear translocation of the XA21 kinase domain (reviewed in [43,44]). The molecular role of XB12 remains unclear.

In this work we identified and cloned the wheat orthologs of rice *RAR1/HSP90/SGT1*, *NPR1-NH1*, *XA21* and *XB12* and their known direct interactors. We then evaluated the degree of conservation in protein-protein interaction in rice and wheat by pair-wise interaction tests using yeast-two hybrid assays (Y2H) and *in vivo* bimolecular fluorescence complementation (BiFC). To facilitate the description of the different interactions the rice genes and protein names will be preceded hereafter by an “r” and the corresponding wheat orthologous genes and proteins by a “w”.

## Results

### The RAR1/SGT1/HSP90 protein complex

The wheat sequences of all the protein components of the RAR1/SGT1/HSP90 complex were previously described and deposited in GenBank (Table 1[45,46]). Genes homologous to *SGT1* and *RAR1* were identified in wheat, with high protein identity levels (82% and 78%, respectively; [43]). These levels of protein identity are within the range usually observed between conserved rice and *Triticeae* orthologous proteins (Table 1, [27]). Rice *SGT1* is located on rice chromosome 1, in a region that is collinear with wheat chromosome 3 [44,45], where the wheat *SGT1* homolog was mapped. A single *SGT1* gene was found in wheat and a reciprocal BLASTP search of the rice predicted proteome using the wheat SGT1 protein sequence results in a single rice match with high similarity (Os01g43540.1 - rSGT1, e^-162^, 82% identity; second best match: Os01g32930.1, 2e^-54^, 44% identity), providing further support that these genes are true orthologs. The phylogenetic relations determined using the Neighbor Joining (NJ) method as implemented in MEGA5 [47] also supports the orthologous relation between the rice and wheat SGT1 proteins (Additional file 1: Figure S1A).

**Table 1:** Rice and wheat orthologous proteins used in this study

**Protein**	**Rice accession**	**Wheat accession**	**Identity**^**1**^	**Similarity **^**1**^	**Description**	**Role in disease resistance in rice**	**Citations**
XA21	AAC49123.1	JX424300	69^2^%	82%^2^	Receptor kinase-like protein	Confers resistance to *Xoo*	[14]
		JX424301	65^2^%	78%^2^			
XB2	NP_001057395.1	JX424303	47%	56%	PHD-finger family protein	-	[4]
XB3	AAK58690.1	JX424304	87%	91%	E3 ubiquitin ligase	Partial positive regulator of *XA21 *resistance to *Xoo*	[19]
XB11	NP_001176613.1	JX424305	84%	90%	C2 calcium/lipid binding domain containing protein	-	[4]
XB12	NP_001065493.1	JX424306	90%	94%	Shikimate kinase-like protein	-	[4]
XB15	NP_001051726.1	JX424307	82%	86%	Phosphatase 2c	Partial positive regulator of *XA21 *resistance to *Xoo*	[6]
XB22	BAG88226.1	JX424308	74%	85%	Tetratricopeptide repeat (TPR) containing protein	-	[4]
XB24	NP_001044383.1	JX424309	70%	76%	ATPase	Negative regulator of *XA21 *mediate resistance to *Xoo*	[48]
XAK1	NP_001052975.1	JX424310	94%	96%	Rice BAK1 homolog (receptor kinase-like protein)	Positive regulator of *XA21 *mediated resistance to *Xoo*	[49]
WRKY76	DAA05141.1	JX424311	67%	77%	WRKY transcription factor	Negative regulator of *XA21 *mediated resistance to *Xoo*	[4]
XB12IP1	AAU44098.1	JX424312	71%	78%	DNA-binding domain (similar to AP2) AP2/ERF domain-containing transcription factor	-	[4]
XB12IP2	NP_001053022.1	JX424313	49%	58%	Sterile alpha motif (SAM) domain family protein	-	[4]
XB12IP5	BAF17190.1	JX424314	78%	87%	Unknown function	-	[4]
NPR1	AAX18700.1	JX424315	81%	88%	*Non-expressor of PR1 *protein	Controls systemic acquired resistance; over-expression enhances resistance to *Xoo*	[16]
TGA2.1	EEC82717.1	JX424316	87%	91%	bZIP transcription factor	Negative regulator of rice basal resistance to *Xoo*	[17,50]
TGA2.2	AAT28674.1	JX424317	93%	97%	bZIP transcription factor	-	[17,50]
TGA2.3	AEF30411.1	JX424318	85%	89%	bZIP transcription factor	-	[17,50]
NRR	AAW80625.1	JX424319	56%	61%	NPR1 interactor	Negative regulator of *XA21 *mediate resistance to *Xoo*	[15]
		JX424320	55%	61%			
NRRH1	NP_001055341.2	JX424321	34%	37%	NPR1 interactor	NRR paralog	[4]
LG2	NP_001044868.1	JX424322	77%	81%	bZIP transcription factor	-	[50]
RAR1	Q6EPW7.2	EF202841.1 [45]	78%	85%	CHORD domain-containing protein	Required for functionality of some R proteins; partial positive regulator of *XA21 *mediated resistance to *Xoo*	[30-33]
HSP90.2	XP_483191	ADF31758.1 [46]	94%	99%	Heat shock protein 90	Required for functionality of some R proteins	[30-33]
HSP90.3	BAD33406	ADF31760.1 [46]	95%	98%	Heat shock protein 90	Required for functionality of some R proteins	[30-33]
SGT1	AAF18438.1	EF546432.1 [45]	82%	89%	Plant ortholog of the yeast cell Cycle regulator SGT1	Required for functionality of some R proteins	[30-33]

^1 ^The proportion of identical and similar amino acid sites were calculated using BLASTP local alignments.

^2 ^For XA21 similarities are computed based on alignments of the cytosolic domains including the C-terminal kinase domain and the juxtamembrane (JM) as in [48] that were used in the protein-protein interaction tests.

*RAR1* is also a single copy gene in wheat and in rice, but unlike *SGT1*, *RAR1* was not found in syntenic regions in the two grasses. The *rRAR1* gene is located on rice chromosome 2 in a region that is syntenic with wheat chromosome 6, not with wheat chromosome 2 where *wRAR1* was found. *RAR1* was mapped in barley on chromosome 2 [31], which is co-linear with wheat chromosome 2 [51]. The wheat - barley synteny at the *RAR1* locus together with the observation that the *B. distachyon RAR1* copy is located on chromosome 3 (*Bradi3g45030.1*; Additional file 1: Table S1), which is syntenic with rice chromosome 2, suggests that the *RAR1* locus change in chromosomes location occurred after divergence between *B. distachyon* and wheat, but before the wheat barley divergence. The NJ phylogenetic tree based on the multiple-alignment of RAR1 protein sequences reflects the evolutionary history of these grass species [52]. These phylogenetic relationships together with the high level of similarity between single copy genes (85% protein similarity) supports the orthologous relationship between the wheat and rice *RAR1* genes in spite of their non colinear location (Additional file 1: Figure S1B).

Three HSP90 genes have been described in wheat, *wHSP90.1*, *wHSP90.2*, and *wHSP90.3*, but only the last two have been implicated in disease resistance [46] and will be discussed in this study. A phylogenetic analysis of the wheat and rice *HSP90* complete cDNAs [46] indicates that the *wHSP90.2* gene located in the short arm of wheat homeologous group 7 is more closely related to the rice gene *rHSP90.2*, which is located in the co-linear rice chromosome 8 (Additional file 1: Table S1; Additional file 1: Figure S1C). The *wHSP90.3* gene located in the long arm of wheat homeologous group 5 is more closely related to the recently duplicated rice genes *rHSP90.3* and *rHSP90.4*, which are both located in the colinear rice chromosome 9 and differ from each other only by a single amino-acid (Additional file 1: Table S1; Additional file 1: Figure S1C [46]).

Full-length coding regions of *wRAR1*, *wSGT1*, *wHSP90.2* and *wHSP90.3* were PCR-amplified from *T. aestivum* cDNA and cloned into *pNLex* and *pLAW10* or *pLAW11* vectors for both LexA and Gal4 based Y2H assays. A LexA based system was previously used to developed the rice immune response interactome [4] and was adopted in this study to test all wheat - rice protein interaction to facilitate direct comparisons with previous rice-rice protein interactions determined using this system [4]. For wheat - wheat protein interactions we implemented the Gal4 based Matchmaker Gold Yeast Two-Hybrid System (Clontech, http://www.clontech.com/). This system includes four independent reporters that allow testing interactions at different stringencies and is being used in our laboratory to establish *ab initio* wheat protein interaction networks using cDNA Y2H library screening (Yang and Dubcovsky, unpublished). By using the same Gal4 system we will be able to seamlessly integrate the interactions observed in this and other studies into our developing wheat interactome map.

**Figure 1:**
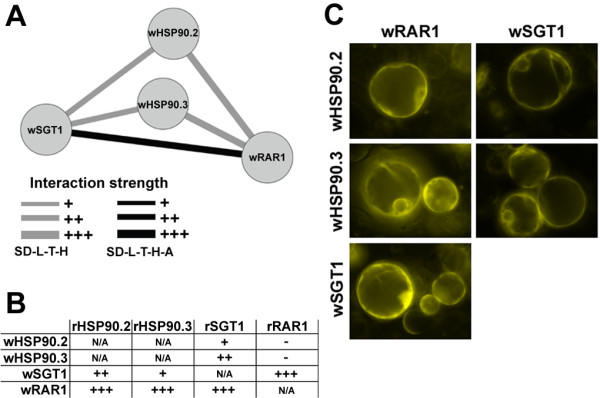
**The RAR1/SGT1/HSP90 protein complex. **(**A**) Schematic representation of the protein-protein interactions in wheat between the components of the RAR1/SGT1/HSP90 protein complex. (**B**) Pair-wise yeast-two-hybrid test of interaction between wheat and rice component of the RAR1/SGT1/HSP90 protein complex. (**C**) Bimolecular fluorescence complementation assay (BiFC) assays showing positive interactions between the wheat components of the RAR1/SGT1/HSP90 protein complex in rice protoplasts. A strong cytosolic fluorescence signal generated by the complemented YFP reporter proteins was observed in all the interactions tested.

We observed a strong interaction between wRAR1 and wSGT1, which was confirmed in media lacking both histidine (-H) and adenine (-A), that results in a more stringent selection pressure (Figure 1A and Additional file 1: Figure S2A). We then tested all possible pair-wise interactions between wRAR1, wSGT1, wHSP90.2, and wHSP90.3 (Figure 1B and Additional file 1: Figure S2B). The two wHSP90 proteins interacted with both wSGT1 and wRAR1 in the absence of H, but not in absence of both A and H. The RAR1-SGT1 and HSP90s-SGT1 interactions were also observed when wheat proteins were tested with the corresponding rice proteins (Figure 1B).

**Figure 2:**
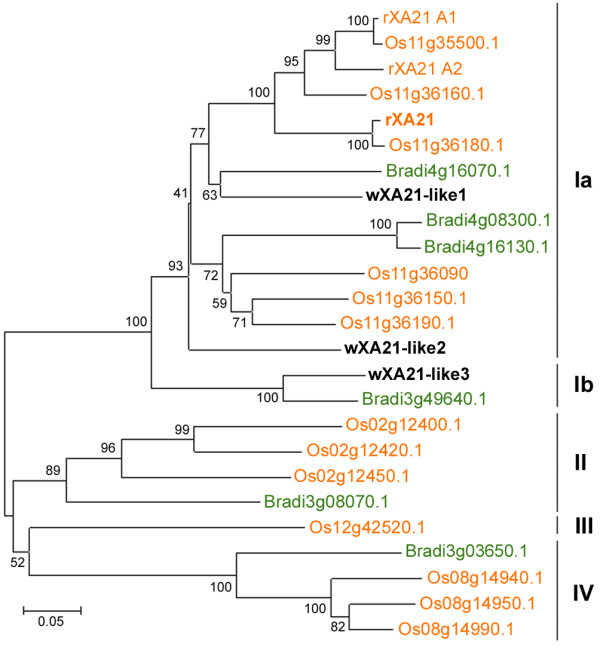
**Phylogenetic tree of *XA21 *homologs. **The represented tree is the bootstrap consensus tree inferred from 1000 replicates generated using the Neighbor-Joining method [53]. The percentage of replicate trees in which the associated sequences clustered together in the bootstrap test (1000 replicates) are shown next to the branches. The analysis involved 25 amino acid sequences and 242 amino acid positions. All positions containing gaps and missing data were eliminated. Evolutionary analyses were conducted in MEGA5 [47]. The *XA21 *clade was divided into 4 subclades as reported in [54].

All five Y2H positive interactions observed between wheat proteins of the RAR1/SGT1/HSP90 complex (Figure 1A) were further confirmed using BiFC assays in rice protoplasts [8]. BiFC allows direct visualization of protein-protein interactions in living plant cells. For all five interactions between wheat proteins of the RAR1/SGT1/HSP90 complex we observed strong positive cytosolic signal in the rice protoplasts generated by the complementation of the yellow fluorescence protein (YFP) (Figure 2C and Additional file 1: Figure S3). No fluorescence was observed when individual wheat proteins were co-transfected into rice protoplasts with empty split YFP vectors in the control experiments (Additional file 1: Figure S3).

### The NPR1 node

The full-length wheat ortholog of rice *NPR1-homolog1* (*NH1*; henceforth *rNPR1*) was isolated from *T. turgidum* ssp. *durum* cDNA. Initial amplification was carried out using oligonucleotides designed to conserved regions identified in the barley NPR1-like protein (AM050559.1). The cloned *wNPR1-like* gene was 86% and 84% identical to the *B. distachyon* (Bradi2g05870.1) and *rNPR1* (AY923983.1) nucleotide sequences, respectively. A phylogenetic analysis of the encoded NPR1 proteins from monocots and dicots was performed using the Neighbor Joining method [47]. The analysis also included sequences from close paralogs, such as the rice NH2-5 copies and the *Arabidopsis thaliana* NPR2 and NPR3 copies (Additional file 1: Figure S4). Wheat NPR1 formed a distinct and well-supported clade (100% bootstrap) with the other graminaceous NPR1 proteins supporting the orthology between wNPR1-like and rNPR1. A larger clade, also well-supported (100% bootstrap), grouped together the monocot and dicot NPR1 sequences. Rice, *B. distachyon*, and wheat NPR1-like genes were also found within syntenic chromosomes further supporting the orthologous relationship between them (Additional file 1: Table S1). The full-length wNPR1-like coding sequence was cloned as bait and tested for its ability to establish protein-protein interactions with known rice rNPR1 interactors (Table 1).

In the available wheat databases we identified orthologous copies of seven of the eight known rice proteins that were shown to interact with rNPR1 (Table 1). Specific members of the TGA family of basic-region leucine zipper (bZIP) transcription factors are known to interact with NPR1 and mediate its function both in rice [50] and Arabidopsis [7,55]. We identified and cloned the wheat orthologs of all three TGAs which interact with rNPR1. Their orthology was confirmed by phylogenetic analysis (Additional file 1: Figure S5) and location in syntenic chromosomes (Additional file 1: Table S1). We also identified two wheat orthologous copies of the rice NRR protein (Negative Regulator of Resistance) that was previously shown to strongly interact with rNPR1 and negatively regulate its activity [15]. The DNA identity between wNRR-like1 and wNRR-like2 is 86%, which is much lower than the average 97% identity expected between genes duplicated by polyploidy (homeologous gene copies; [56]). This result suggests that these two wNRR genes are paralogous copies. Based on their position in the phylogenetic tree (Additional file 1: Figure S5) and the absence of multiple *NRR* copies in the complete genomes of rice and *B. distachyon*, the *wNRR-like1* and *wNRR-like2* appear to be the result of a recent duplication event that occurred in the Triticeae lineage. Without a complete barley genome it was not possible to determine if the *NRR-like 2* homologue in barley was deleted or simply not available in current databases. Rice rNPR1 was also shown to interact with three rNRR paralogs (rNRRH1-3) and a bZIP transcription factor similar to the maize protein liguless gene (*LG2*[4,50]). Among these other interactors, we could identify and isolate the wheat orthologs of *rNRRH1* and *rLG2* (Additional file 1: Figure S5).

**Figure 3:**
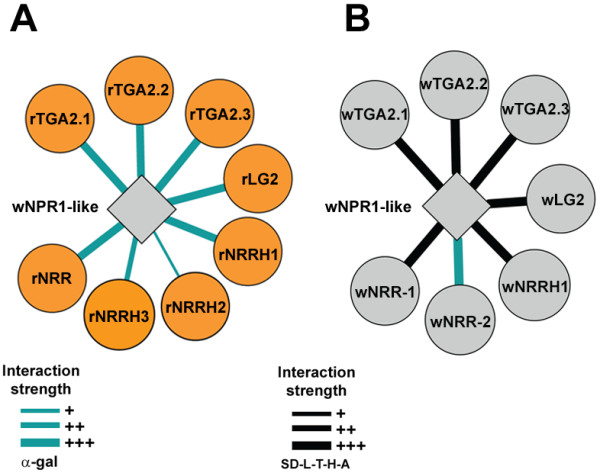
**The wheat NPR1-like protein node. **Schematic representation of protein-protein interactions between the wheat NPR1-like protein and the known interactors of NPR1 in rice (**A**) and their orthologous copies in wheat (**B**).

wNPR1-like showed strong interactions with all the eight known rice rNPR1-interacting proteins, with the exception of rNRRH2, for which a weaker interaction was observed (Figure 3; Additional file 1: Figure S6A). The three *wTGAs* were PCR-amplified from cDNA of *T. monococcum*, whereas the other four genes were synthesized using different templates: *wNRR-like1* and *wNRR-like2* used *T. aestivum* ESTs DR738016.1 and FGAS026833, while *wNRRH1* and *wLG2* used *de novo* transcriptome assemblies from diploid wheat *T. urartu* (K. Krasileva and J. Dubcovsky, unpublished) and the sequences where deposited in GenBank (Table 1 for accession numbers). All seven wheat genes were cloned in the yeast prey vector and were shown to interact strongly with wNPR1-like (Figure 3; Additional file 1: Figure S6B). All Y2H interactions between wNPR1 and wheat proteins, with the exception of wLG2, were also confirmed in rice protoplasts using the BiFC system where sharp fluorescent signals produced by the YFP reporter protein were detected in the protoplast nuclei (Additional file 1: Figure S7).

### The XA21 node

Using the amino acid sequence of rice XA21 (rXA21; AAC49123.1) as a query for TBLASTN searches of the publicly available wheat sequence databases, we identified two potential orthologous copies in the wheat genome (AY072046.1 and HP619392.1; [29]). The two partial sequences were used as a template to clone the full-length wheat *XA21-like* genes (*wXA21-like1* and *wXA21-like2*; Table 1) from *T. aestivum*. The kinase domain of wXA21-like1 and wXA21-like2 showed 71% and 66% amino-acid identity and 82% and 78% similarity to the kinase domain of rXA21, respectively. An additional and more distant homolog of rXA21 was identified in the *Ae. tauschii* genome (*wXA21-like3*). This third copy shares slightly lower similarity to rXA21 than wXA21-like2 (62% identity and 71% similarity in the kinase domain)*.*

To further determine the orthologous relationship between *rXA21* and the identified wheat genes, the amino-acid sequences of the wheat *XA21-like* genes were aligned to rXA21 and other known XA21 homologs in rice and *Brachypodium distachyon*, and a phylogenetic tree was constructed (Figure 2). XA21 proteins from grass species (without wheat sequences) were previously divided into 4 major clades, with clade 1 including rXA21 together with its closest rice paralogs and *B. distachyon* homologs [54]. The tree resulting from our phylogenetic analysis is consistent with the previous report (Figure 2) with *clade 1* and *clade 2* supported by high bootstrap values of 100% and 89%, respectively. Both wXA21-like copies were part of *clade 1a*, whereas wXA21-like3 clustered together with the *B. distachyon* protein Bradi3g49640.1 within the *clade 1b* (Figure 2). The location of *wXA21-like1* on chromosome 5 is syntenic with rice chromosome 11 that carries *rXA21* (Additional file 1: Table S1), which provides further support for the orthologous relationship between *wXA21-like1* and *rXA21*. However, because of their phylogenetic position within the same clade and their similar identity to rXA21, both *wXA21-like1* and *2* genes were considered potential orthologous copies of *rXA21* and were both used to analyze the degree of conservation of the protein-protein interactions in rice and wheat.

Both the C-terminal kinase domain and the JM domain of XA21 have previously been shown to serve as high affinity binding sites for downstream signaling proteins [6,57]. Therefore, the entire cytosolic portion of rXA21 spanning both JM and kinase domain and comprising 348 amino-acids was previously used as bait for the library screens that resulted in the development of the XA21-centered interactome [4,6]. The same truncated forms of the rice and wheat XA21 proteins were used in this study.

**Figure 4:**
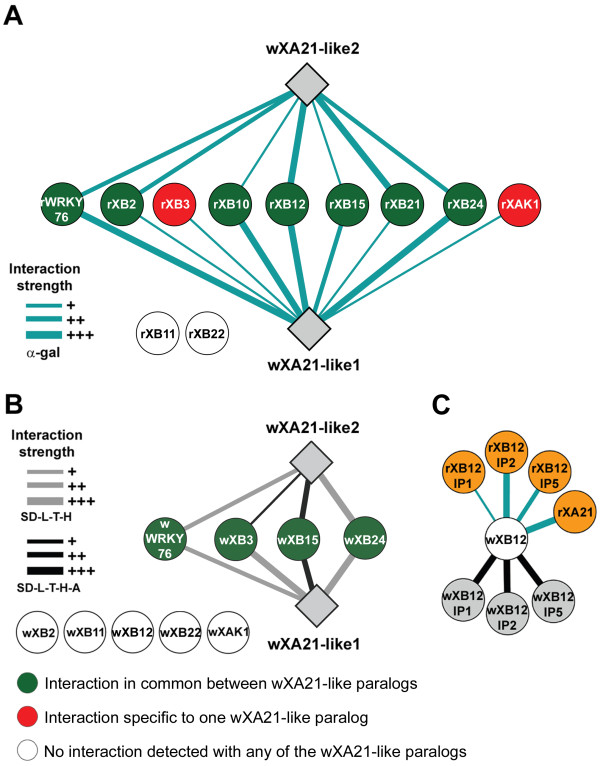
**The wheat XA21-like protein node. **Schematic representation of protein-protein interactions between the two wheat XA21-like proteins and the known interactors of XA21 in rice (**A**) and their orthologous copies in wheat (**B**). (**C**) shows the interaction between wXB12 and rice and wheat XB12 interacting proteins (XB12IPs).

Protein-protein interactions between wXA21-like proteins and the known protein interactors of rXA21 (XA21 binding proteins; henceforth designated XBs) were tested using the same LexA based yeast two hybrid assay (Y2H) as was used to test the rice-rice protein interactions [4,48]. The cytosolic domains of both wXA21-like genes were cloned into the Gateway-compatible vector pNLex as baits. Y2H pB42AD prey vectors carrying 11 different rice XBs were available [4]. Of the 11 wheat-rice interactions tested, wXA21-like1 interacted with 9 of them. Four of these interactions were strong (rWRKY76, rXB10, rXB12, rXB24) and the other five were weaker (rXB2, rXB3, rXB15, rXB21, and rXAK1). wXA21-like2 interacted with seven rXB proteins , including the four showing strong interactions with wXA21-like1 and rXB2 and rXB21 (Figure 4A; Additional file 1: Figure S8). None of the two wheat wXA21 proteins interacted with rXB11 and rXB22.

Out of the 11 known interactors of XA21 in rice, we were able to isolate nine putative orthologous wheat copies. Wheat orthologs of *rXB10* and *rXB21* were not identified. Partial sequences of the wheat homologous genes of rice *XBs* were identified in the publicly available EST collection (GenBank and in the 454 sequence reads of the hexaploid wheat genome (http://www.cerealsdb.uk.net/). Chromosome location (Additional file 1: Table S1), sequence similarity (Table 1) and phylogenetic relationship between the rice, *B. distachyon* and wheat copies (Additional file 1: Figure S9), supported the orthologous relationship between the cloned wheat genes and their rice homologs. Orthologous relationships were confirmed for all wheat-rice orthologous protein pairs by reciprocal BLASTX searches (i.e.: rice and wheat orthologs were each other’s best match in their respective genomes).

The two *wXA21-like* genes and the wXB orthologs were PCR-amplified from cDNA of *T. aestivum* and *T. monococcum*, respectively*.* The *wXA21-like1* and *wXA21-like2* genes were cloned in yeast vectors both as prey (*pLAW11*) and bait (*pLAW10*)*.* Most *wXB* orthologs were cloned in the yeast prey vector, with the exception of *wXB2, wXB3*, *wXB12* and *wXB15* that showed auto-activation when cloned in this vector. These last four wheat genes were cloned as baits and tested with *wXA21-like1* and *wXA21-like2* proteins cloned as preys (Additional file 1: Figure S8). Both wXA21-like1 and wXA21-like2 showed a strong interaction with wXB15 and weaker interactions with wXB3, wXB24 and wWRKY76 (Figure 4B). As observed with rXB11, none of the wXA21-like paralogs interacted with wXB11, but unlike what was observed with rice XA21-XB interactions, we did not detect interactions for both wXA21-like paralogs with wXB2, wXB22, and wXAK. Since some WRKY proteins have been shown to be nuclear transcription factors connecting disease resistance pathways [58], we tested if wNPR1 was able to interact with wWRKY76. We observed a strong interaction of wWRKY76 with wNPR1 both in -H and –H-A selection media (Additional file 1: Figure S6D).

To further explore the conservation between wheat and rice protein interactions we tested a secondary node of the rXA21 interactome. We selected the *rXB12* node because its function is still unknown due to the lethality of the *XB12* silencing in rice (Chern, Chen and Ronald, unpublished). Putative wheat orthologs of three known rice interactors of *rXB12* (*rXB12-IP1, rXB12-IP2*, and *rXB12-IP5*) were isolated from *T. turgidum* ssp. *durum* cDNA (Table 1; Additional file 1: Figure S10). The wheat wXB12 protein showed strong interaction with rice rXA21, but no detectable interactions with the two wXA21-like proteins. The wXB12 protein also showed strong interactions with wXB12-IP1, wXB12-IP2, and wXB12-IP5 and with the corresponding proteins from rice (Figure 4B). In rice, rXB12 was shown to interact with the RAR1/SGT1/HSP90 protein complex through a direct association with rHSP90 [4]. We tested the interaction of wXB12 with all the rice and wheat components of the RAR1/SGT1/HSP90 protein complex. Only a very weak interaction between wXB12 and both wheat and rice SGT1 was observed (Additional file 1: Figure S2), confirming the association of XB12 with the protein complex, although through the association with a different interactor.

Wheat Y2H interactions detected for the XA21 and XB12 nodes were further validated using BiFC rice protoplast (Additional file 1: Figure S11). Both *wXA21-like* genes were cloned in frame to the C-terminal fraction of the yellow fluorescent protein [8], whereas *wWRKY76*, *wXB3*, *wXB15*, *wXB22*, and *wXB24* were all fused to the N-terminal fraction of the YFP protein. In rice protoplast interactions were detected for only a few of the pair-wise BiFC tests carried out (Additional file 1: Figure S11). We observed weak cytosolic and nuclear interactions between XB24 and both wXA21-like proteins, and between wXB15 and wXA21-like1 (Additional file 1: Figure S12). In rice protoplasts we also detected the interaction between wXB12 and wXB12IP5 (Additional file 1: Figure S12).

## Discussion

To determine the degree of conservation of the interactions between proteins involved in defense responses in wheat and rice, we identified and cloned the wheat orthologs of rice genes encoding proteins involved in four well-characterized nodes of the rice interactome and tested their protein-protein interactions using both rice-wheat and wheat-wheat protein pairs. Despite more than 50 million years of divergence time from their common ancestor, we observed an extensive conservation in the interactions of these proteins in rice and wheat. In total we tested 86 binary protein interactions using Y2H assays; 48 interactions were tested between rice and wheat proteins and 38 between wheat proteins. Eighty three percent of the known interactions were confirmed between wheat proteins and rice interactors and 76% were confirmed using wheat protein pairs.

### Orthologous relationships between wheat and rice gene pairs

The identification of orthologous gene pairs is the first critical step for a reliable comparison of protein interactions [59]. The availability of comprehensive wheat EST databases, 454 sequencing reads, and the complete annotated genome of the close relative *B. distachyon* greatly facilitated this task. The reciprocal highest sequence similarity between genes in two organisms is a valid indication of orthologous relations between genes [52,60]. The knowledge of syntenic relationships between rice, *B. distachyon*, and wheat chromosomes provided an additional parameter to establish orthology [60,61]. Among all the wheat orthologs identified in this study, a lack of co-linearity between rice, *B. dystachyon*, and wheat was found only for RAR1. This gene, however, was co-linear in barley and wheat suggesting that the chromosome rearrangement for this *locus* occurred after the divergence between *B. distachyon* and the wheat-barley lineage [52]*.*

We identified and included in this study 24 wheat orthologs out of the 28 targeted rice proteins targeted in this study. Despite the absence in the currently available databases of wheat orthologs for *NRRH2*, *NRRH3*, *XB10*, *XB21,* we cannot rule out the existence of functional copies of these genes in the wheat genome since the sequence of the complete wheat genome is not yet available. In those cases where we observed gene duplication events that occurred in wheat after the divergence between rice and wheat, such as for the wheat copies of *rXA21* and *rNRR*, we included the available wheat paralogs in the analysis. Only once a high quality and fully annotated wheat genome becomes available it will be possible to determine if the wheat *NRRH2*, *NRRH3*, *XB10*, *XB21* orthologs are present and if there are additional wXA21 and wNRR paralogs.

### Complete network conservation in the RAR1/SGT1/HSP90, NPR1, and XB12 nodes

The complete conservation of the interactomes for the RAR1/SGT1/HSP90 and NPR1 nodes likely reflects the importance of their functions in the immune responses across the plant kingdom. It has been demonstrated that both nodes play critical roles in the integration of signaling pathways in multiple plant species to direct defense responses against a broad range of pathogens (reviewed in [62-64]). While a role of XB12 and its interactors in the rice defense response has yet to be defined, the lethal phenotype resulting from its down regulation indicates that this gene is essential for rice survival (Chern, Chen, Seo and Ronald, unpublished). The essential role of XB12 may explain the complete conservation of the interactions centered on XB12 tested in this study.

In cereals the RAR1/SGT1/HSP90 molecular chaperone is known to be required for the resistance mediated by many NBS-LRR resistance genes such as barley *Mla6* and *Mla12*, effective against powdery mildew [30,64,65], wheat *Lr21*, effective against leaf rust [38] and the rice *XA21* pattern recognition receptor [4]. The functional centrality of this node extends across the entire plant kingdom. For example, in Arabidopsis, perturbations of this complex resulted in higher sensitivity to bacterial and viral pathogens [32,66-69]. Importantly, this functional conservation is also associated with the retention of protein-protein interaction specificities even between monocots and dicots as suggested by the observation that barley RAR1 can interact with two recently duplicated Arabidopsis SGT1 paralogs [30].

NPR1 functions as a central regulator of salicylic acid-mediated systemic acquired resistance, the induction of systemic resistance by *Rhizobacterium* spp., and the interaction between defense signaling pathways [62]. Many pathogen proteins from evolutionary distant pathogens target a limited set of highly connected hubs to disrupt the normal deployment of immune responses [11]. Sequence diversity between NPR1 alleles in Arabidopsis suggests strong positive selection imposed by pathogen pressure compatible with an “arms race” model of evolution, further supporting a critical position of NPR1 in the immune response network [70]. The NPR1 signaling pathway was demonstrated to be conserved between rice and Arabidopsis [50]. Our study extends this observation to wheat. Although NPR1 function remains to be mechanistically linked to disease resistance in wheat, the observation that benzothiadiazole (BTH) treatments induce acquired resistance in wheat and enhance resistance to powdery mildew suggests that NPR1 may play a similar role in wheat as observed in other plant species [62].

### Interaction differences between rice and wheat in the XA21 node

All the interaction differences detected in this study between rice and wheat were observed in the XA21 node, where 66% of the interactions between rice proteins involved in this node were not detected between the corresponding wheat homologs. This lower conservation might be related to the different duplication history of the *XA21* gene family in the wheat and rice lineages. Although *XA21* duplications were found in the wheat – *B. distachyon* lineage, the degree of expansion appears to be much greater in rice than in wheat and other grasses [54]. Recombination events at conserved DNA sequences between paralogs, large sequence duplications, and transposable element insertions have contributed to the amplification and diversification of the *rXA21* gene family [71]. An analogous larger expansion in rice than other plant species was also demonstrated for the RLP genes (a receptor like proteins lacking the cytosolic kinase) [72]. It is tempting to speculate that the higher duplication levels of XA21 in rice may be related with its particular growth conditions in standing water and its subtropical distribution, which might increase the pressure of bacterial pathogens. To test if the wXA21-like copies acquired novel interactors in wheat we have initiated the Y2H screening of wheat cDNA libraries.

A higher positive selection on proteins involved in biotic stress responses may accelerate the divergence of the related protein-protein interactions. In *Drosophyla melanogaster*, *Saccaromyces cerevisiae, Caenorhabditis elegans* and *Homo sapiens* significantly faster rates of changes in protein-protein interactions were observed in network nodes involved in immune and abiotic stress responses [73]. In particular, proteins that are part of intracellular signaling cascades were about two-fold more likely to show altered protein interactions than proteins involved in primary metabolism. A possible explanation is that these proteins involved in signaling cascades carry domains that can interact with diverse and common structural motifs and, thus, are more efficient in gaining novel interactors [73].

## Conclusions

In conclusion, the high conservation of protein-protein interactions centered on key regulators of the rice defense response suggests that the existing experimentally generated rice interactome is a useful initial predictor of wheat protein interactions. Our results also point to a higher degree of interaction divergence in those interactome nodes that show divergent duplication history, perhaps related to selective pressure by species-specific pathogen types. A genome-wide search of wheat orthologs to the rice genes included in the stress response interactome can be used to generate a predicted wheat interactome. The hypothetical interactions will need to be validated experimentally and be complemented with wheat cDNA library screens to identify wheat-specific protein interactions. Finally, available wheat TILLING mutant populations [74] or transgenic approaches can be used to validate the biologically relevance of genes coding for protein located at nodes of the wheat stress interactome.

## Methods

### Plant material

*Triticum monococcum* cv. DV92 was used to isolate all *wXBs*, *wBAK1*, *wWRKY76*, and *wTGAs*; *T. turgidum* ssp. *durum* cv. Langdon was used to isolate *wXB12IPs*, *wHSP90s*, *wSGT1*, *wRAR1*, and *wXB12*; *T. aestivum* was used to isolate *wXA21s*, *wNRRs*; *T. urartu* was used to isolate *wLG2* and *wNRRH1*. All plants were grown in a greenhouse at 20–25°C under long-day photoperiod (8 h of dark/16 h of light) for 4 weeks before leaves were harvested for RNA extraction. The coding sequences of all wheat genes cloned in this study have been deposited in GenBank under accession numbers JX424300-JX424321 (Table 1). The isolation of the rice clones used in this study was described previously [4,6,15,16,19,50].

### Protein-protein interaction assays

Yeast vectors pLAW10 (DNA-binding domain, BD) and pLAW11 (activation domain, AD) and yeast strain Y2HGold were used in the two-hybrid assays to test the interactions between wheat proteins (Clontech, http://www.clontech.com/). pLAW10 and pLAW11 are Gateway (Invitrogen) compatible modifications of the yeast vectors pGBKT7 and pGADT7, respectively, and were a generous gift of Dr. Richard Michelmore (Perroud and Michelmore, unpublished). The lithium acetate method was used for yeast transformation. Transformants were selected on SD medium lacking leucine (-L) and tryptophan (-T) plates and re-plated on SD medium lacking -L, -T, histidine (-H) and adenine (-A) to test the interactions.

Interactions between wheat and rice proteins were tested using a LexA system. BD and AD Gateway compatible vectors pNLex and pB42AD were co-transformed into yeast EGY48/p8op-lacZ (Clontech) by using the Frozen-EZ yeast transformation II kit (Zymo Research). Transformed yeast cells were initially placed on SD media (SD/-His, -Ura, -Trp) and then patched to SD induction media to assay LacZ activity.

For bimolecular fluoresence complementation (BiFC) assays, each set of wheat proteins was recombined into the Gateway compatible pY736 and pY735 vectors to generate YFP-N-terminal fragment and YFP-C-terminal-fragment fusion proteins. Rice protoplasts were prepared, transfected and visualized as described in [75].

## Competing interest

The authors declare that they have no competing interests.

## Authors’ contributions

Conceived and designed the experiments: DC, RR, MSC, PCR, and JD. Performed the experiments: DC, BJY, VM, RR, KL, and DF. Analyzed the data: DC, RR, BY, MSC, and JD. Wrote the paper: DC, JD. All authors read and approved the final manuscript.

## Supplementary Material

Additional file 1: Figure S1 Phylogeny of SGT1 (A), RAR1 (B), and HSP90 (C) homologs. **Figure S2.** Yeast-two-hybrid tests of interactions between components of the RAR1/SGT1/HSP90 protein complex and between XB12 and RAR1/SGT1/HSP90 and XB12IPs. **Figure S3.** BiFC assays showing positive interactions between the wheat components of the RAR1/SGT1/HSP90 protein complex in rice protoplasts. **Figure S4.** Phylogeny of NPR1 homologs. **Figure S5.** Phylogeny of TGA (A), LG2 (B), and NRR (C) homologs. **Figure S6.** Yeast-two-hybrid tests of interactions between the wheat orthologous copy of NPR1 (wNPR1-like) and known rice NPR1 interacting proteins (A) and their orthologous copies in wheat (B & C). In (D): interaction tests between positive wXA21-like1 interacting wXBs and wNPR1. **Figure S7.** BiFC assays showing positive interactions localized in the nuclei between wheat NPR1 and wheat TGAs and NRRs in rice protoplasts. **Figure S8.** Yeast-two-hybrid tests of interactions between the cytosolic domain of wheat XA21 copies (wXA21-like1 & wXA21-like2) and known XA21 interacting proteins (A) and their orthologous copies in wheat (B-D). **Figure S9.** Phylogeny of XA21 interacting proteins. **Figure S10.** Phylogeny of XB12 interacting proteins (wXB12IPs). **Figure S11.** BiFC assays showing positive interactions between wheat XA21-like proteins and wheat XBs in rice protoplasts. **Figure S12.** BiFC assays showing positive interactions between wheat XB12 and wheat XB12IP5 in rice protoplasts. **Table S1.** Chromosome locations and putative synteny of the genes used in this study in rice, *B. distachyon* and wheat. Click here for file
