# Supplementary material for: Comparative analysis of protein-protein interactions in the defense response of rice and wheat
Source: BMC Genomics. 2013 Mar 12;14:166. doi: 10.1186/1471-2164-14-166 (PMC3602203; doi:10.1186/1471-2164-14-166)
Supplement: Additional file 1: Figure S1 — Phylogeny of SGT1 (A), RAR1 (B), and HSP90 (C) homologs. Figure S2. Yeast-two-hybrid tests of interactions between components of the RAR1/SGT1/HSP90 protein complex and between XB12 and RAR1/SGT1/HSP90 and XB12IPs. Figure S3. BiFC assays showing positive interactions between the wheat components of the RAR1/SGT1/HSP90 protein complex in rice protoplasts. Figure S4. Phylogeny of NPR1 homologs. Figure S5. Phylogeny of TGA (A), LG2 (B), and NRR (C) homologs. Figure S6. Yeast-two-hybrid tests of interactions between the wheat orthologous copy of NPR1 (wNPR1-like) and known rice NPR1 interacting proteins (A) and their orthologous copies in wheat (B & C). In (D): interaction tests between positive wXA21-like1 interacting wXBs and wNPR1. Figure S7. BiFC assays showing positive interactions localized in the nuclei between wheat NPR1 and wheat TGAs and NRRs in rice protoplasts. Figure S8. Yeast-two-hybrid tests of interactions between the cytosolic domain of wheat XA21 copies (wXA21-like1 & wXA21-like2) and known XA21 interacting proteins (A) and their orthologous copies in wheat (B-D). Figure S9. Phylogeny of XA21 interacting proteins. Figure S10. Phylogeny of XB12 interacting proteins (wXB12IPs). Figure S11. BiFC assays showing positive interactions between wheat XA21-like proteins and wheat XBs in rice protoplasts. Figure S12. BiFC assays showing positive interactions between wheat XB12 and wheat XB12IP5 in rice protoplasts. Table S1. Chromosome locations and putative synteny of the genes used in this study in rice, B. distachyon and wheat. [file 1471-2164-14-166-S1.pdf]

Comparative analysis of protein-protein interactions in the defense response of rice and wheat

Additional File 1 -

Figure S1 - Phylogeny of SGT1 (A), RAR1 (B), and HSP90 (C) homologs.

Figure S2 - Yeast-two-hybrid tests of interactions between components of the RAR1/SGT1/HSP90 protein complex and between XB12 and RAR1/SGT1/HSP90 and XB12IPs.

Figure S3 - BiFC assays showing positive interactions between the wheat components of the RAR1/SGT1/HSP90 protein complex in rice protoplasts.

Figure S4 - Phylogeny of NPR1 homologs.

Figure S5 - Phylogeny of TGA (A) and NRR (B) homologs.

Figure S6 - Yeast-two-hybrid tests of interactions between the wheat orthologous copy of NPR1 (wNPR1-like) and known rice NPR1 interacting proteins (A) and their orthologous copies in wheat (B & C). In (D): interaction tests between positive wXA21-like1 interacting wXBs and wNPR1.

Figure S7 - BiFC assays showing positive interactions localized in the nuclei between wheat NPR1 and wheat TGAs and NRRs in rice protoplasts.

Figure S8 - Yeast-two-hybrid tests of interactions between the cytosolic domain of wheat XA21 copies (wXA21-like1 & wXA21-like2) and known XA21 interacting proteins (A) and their orthologous copies in wheat (B-D).

Figure S9 - Phylogeny of XA21 interacting proteins.

Figure S10 - Phylogeny of XB12 interacting proteins (wXB12IPs).

Figure S11 - BiFC assays showing positive interactions between wheat XA21-like proteins and wheat XBs in rice protoplasts.

Figure S12 - BiFC assays showing positive interactions between wheat XB12 and wheat XB12IP5 in rice protoplasts.

Table S1 - Chromosome locations and putative synteny of the genes used in this study in rice, *B. distachyon* and wheat.

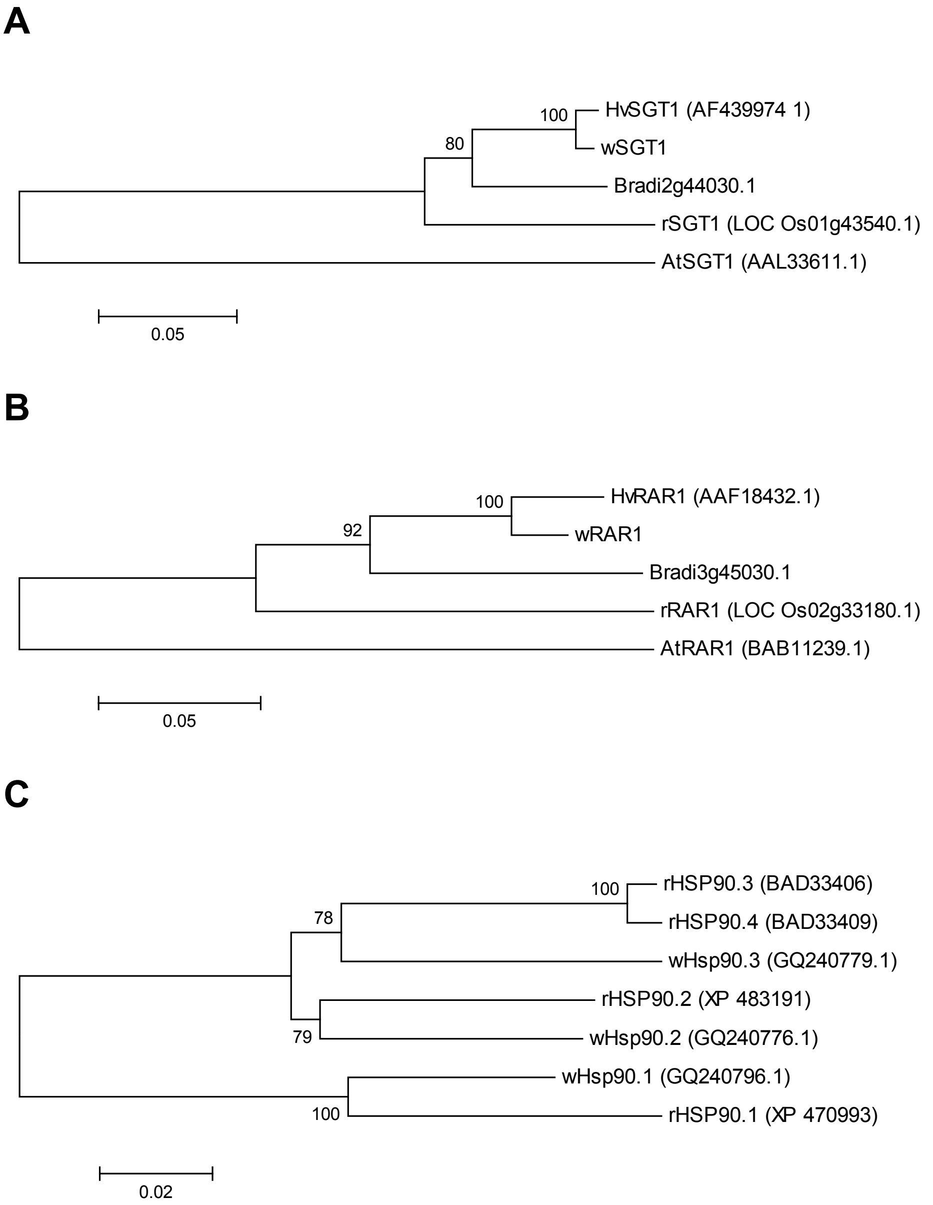

Figure S1 - Phylogeny of SGT1 (A), RAR1 (B), and HSP90 (C) homologs. All represented trees are the bootstrap consensus trees inferred from 1000 replicates generated using the Neighbor-Joining method [73]. The percentage of replicate trees in which the associated sequences clustered together in the bootstrap test (1000 replicates) are shown next to the branches. Multiple alignment of protein sequences was used to infer phylogenetic relations for SGT1 and RAR1. Phylogeny of HSP90 instead was derived from multiple alignment of nucleotide sequences because of the lack of a sufficient number of variable sites between HSP90 protein sequences (Table 1). All positions containing gaps and missing data were eliminated. Evolutionary analyses were conducted in MEGA5 [46].

A

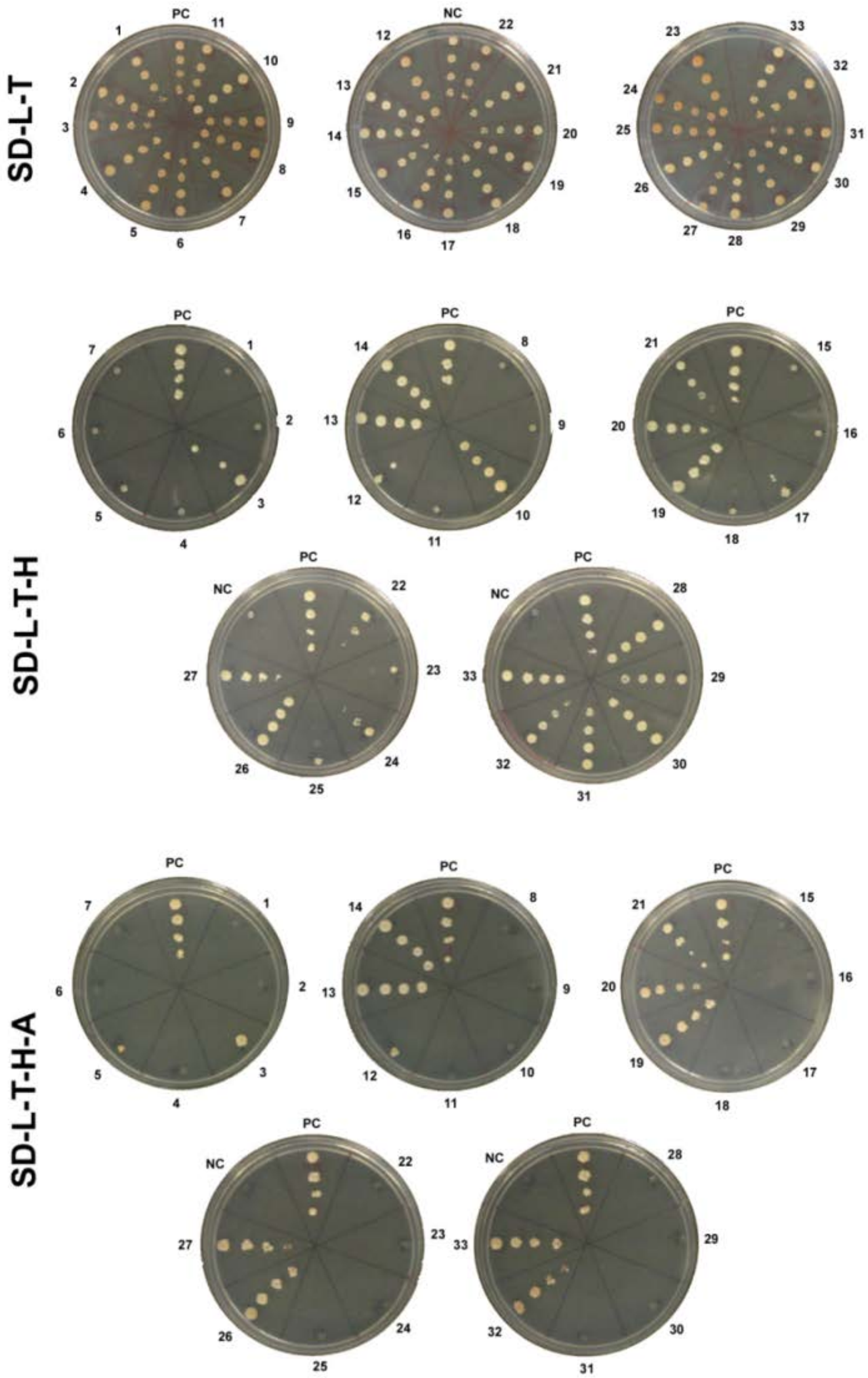

B

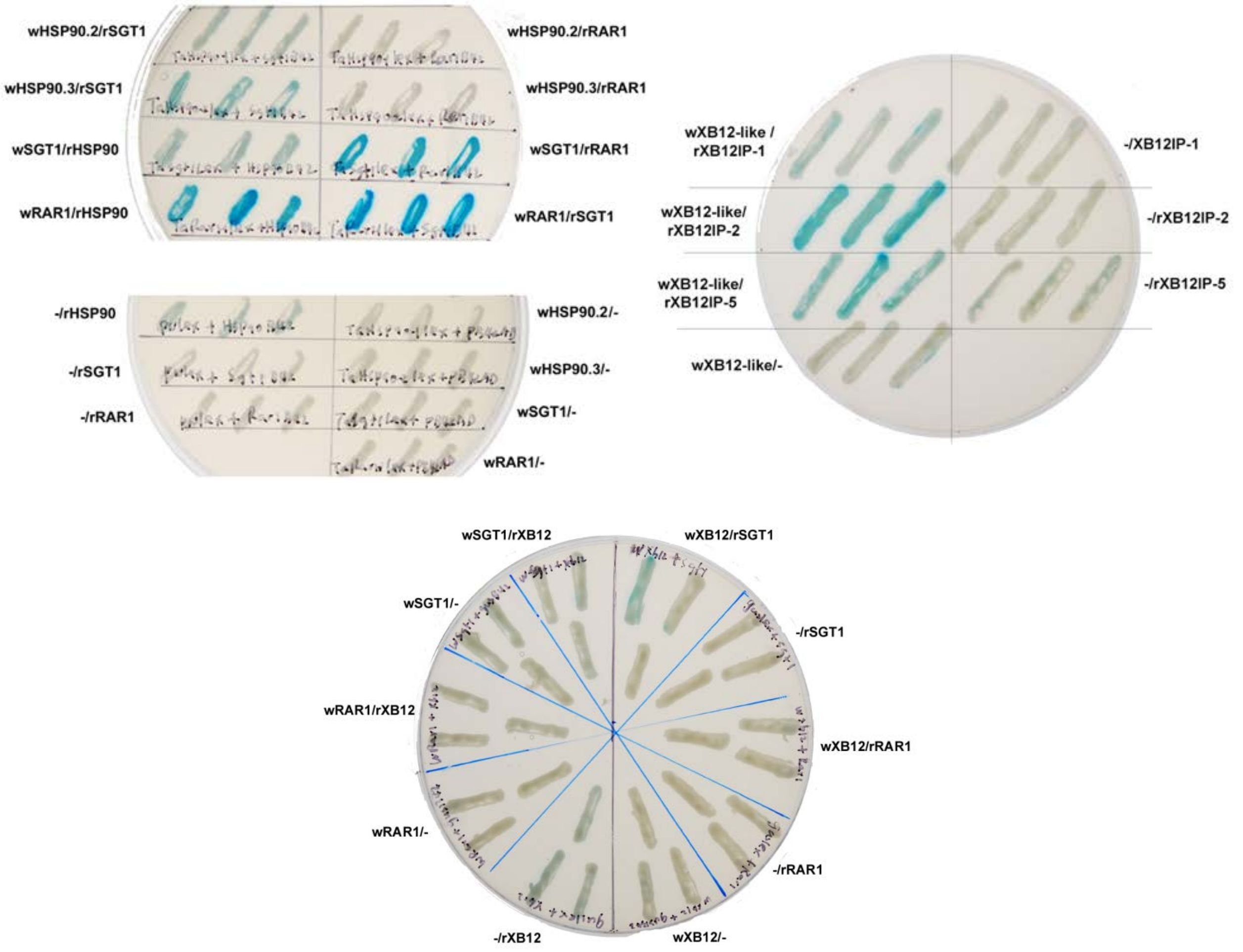

Figure S2 - Yeast-two-hybrid tests of interactions between components of the RAR1/SGT1/HSP90 protein complex and between XB12 and RAR1/SGT1/HSP90 and XB12IPs.

(A) Wheat-wheat interactions: Baits are expressed in the *pLAW10*-BD vector and preys in the the *pLAW11*-AD vector. Positive co-transformation in the Gal4 based system was tested in SD-L-T media, whereas positive interactions were tested in SD-L-T-H and SD-L-T-H-A median, in absence of histidine (-H) and both histidine and adenine (-H-A), respectively. Autoactivation of clones was tested using empty bait (-/) or pray vectors (/-). Numbers in the figure correspond to the following pair-wise Y2H tests: 1) wHSP90.3/-; 2) wHSP90.2/-; 3) wSGT1/-; 4) wRAR1/-; 5) wXB12IP2/-; 6) wXB12IP5/-; 7) wXB12IP1/-; 8) -/wHSP90.3; 9) -/wHSP90.2; 10) -/wSGT1; 11) -/wRAR1; 12) -/wXB12IP2; 13) -/wXB12IP5; 14) -/wXB12IP1; 15) wHSP90.3/wXB12; 16) wHSP90.2/wXB12; 17) wSGT1/wXB12; 18) wRAR1/wXB12; 19) wXB12IP2/wXB12; 20) wXB12IP5/wXB12; 21) wXB12IP1/wXB12; 22) wHSP90.3/wSGT1; 23) wHSP90.3/wRAR1; 24) wHSP90.2/wSGT1; 25) wHSP90.2/wRAR1; 26) wSGT1/wRAR1; 27) wRAR1/wSGT1; 28)

wHSP90.3/wSGT1; 29) wHSP90.3/wRAR1; 30) wHSP90.2/wSGT1; 31) wHSP90.2/wRAR1; 32) wSGT1/wRAR1; 33) wRAR1/wSGT1; PC (positive control); NC (negative control). Interactions between rHSP90 and wXB12 are not shown.

(B) Wheat-rice interactions: Baits are fused to the LexA protein in the *pNLex*-BD vector and preys fused to the B42AD protein in the *pB42AD* vector as prey. Blue colors indicate positive interactions.

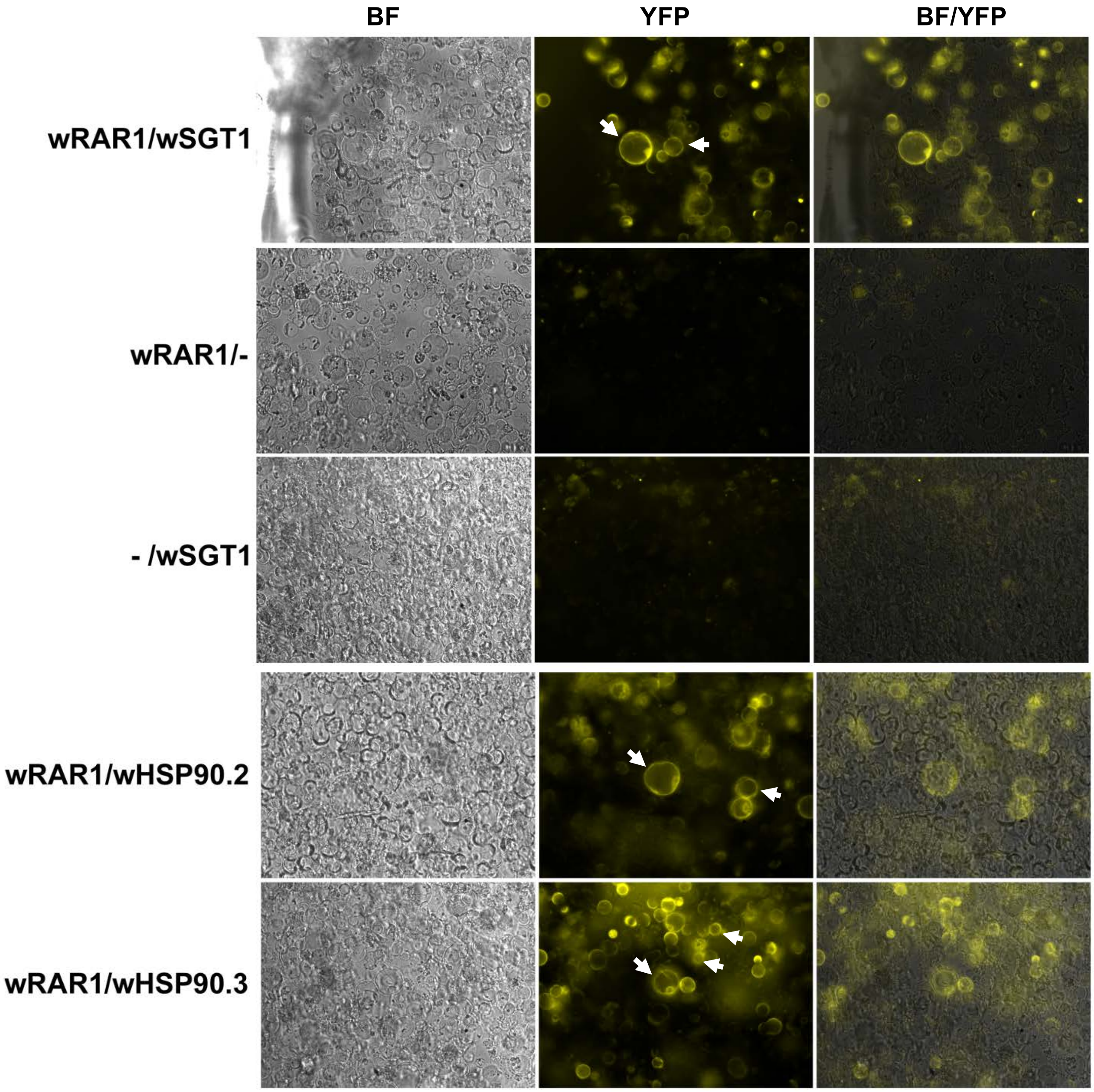
BF
YFP
BF/YFP
wRAR1/wSGT1
wRAR1/-
- /wSGT1
wRAR1/wHSP90.2
wRAR1/wHSP90.3

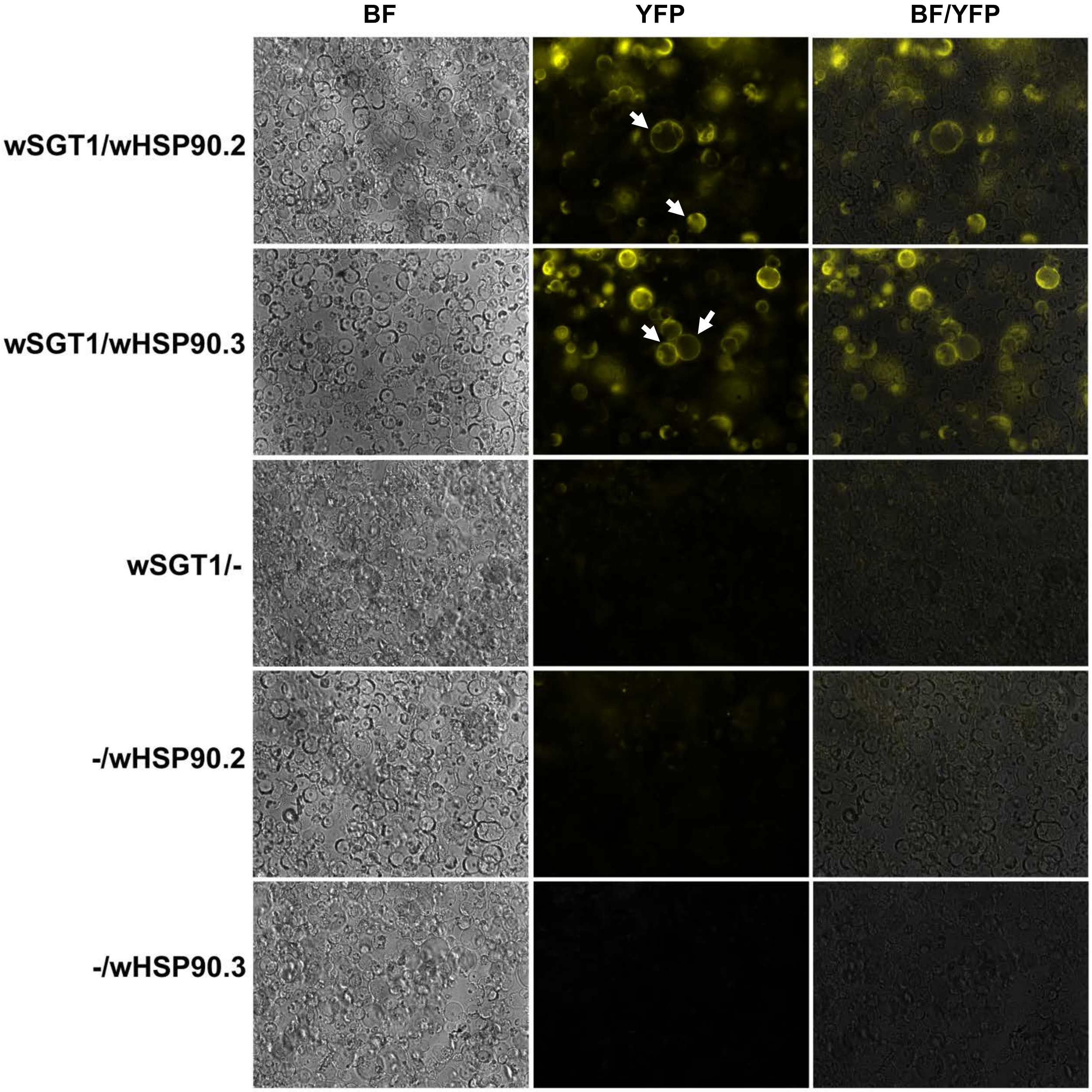

Figure S3 - BiFC assays showing positive interactions between the wheat components of the RAR1/SGT1/HSP90 protein complex in rice protoplasts. Bimolecular fluorescence complementation assay (BiFC) was used to visualize protein-protein interactions in rice protoplasts (arrowhead = examples of positive YFP signal). Images were taken using a Zeiss Axiovert 25 microscope with a 40× objective under either bright-field illumination (BF) or a YFP specific filter (YFP). Unfused YFPN-constructs were used as negative controls. The construct used are as follows: The construct used are as follows: **wRAR1/wSGT1** (735-YC-wRAR1 + 736-YN-wSGT1); **wRAR1/-** [735-YC-wRAR1 + 736-YN-empty (control)]; **-/wSGT1** [735-YC-empty (control) + 736-YN-wSGT1]; **wRAR1/wHSP90.2** (735-YC-wRAR1 +

736-YN-wHSP90.2); **wRAR1/wHSP90.3** (735-YC-wRAR1 + 736-YN-wHSP90.3); **wSGT1/wHSP90.2** (735-YC-wSGT1 + 736-YN-wHSP90.2); **wSGT1/wHSP90.3** (735-YC-wSGT1 + 736-YN-wHSP90.3); **wSGT1/-** [735-YC-wSGT1 + 736-YN-empty (control)]; **-/wHSP90.2** [735-YC-empty (control) + 736-YN-wHSP90.2]; **-/wHSP90.3** [735-YC-empty (control) + 736-YN-wHSP90.3].

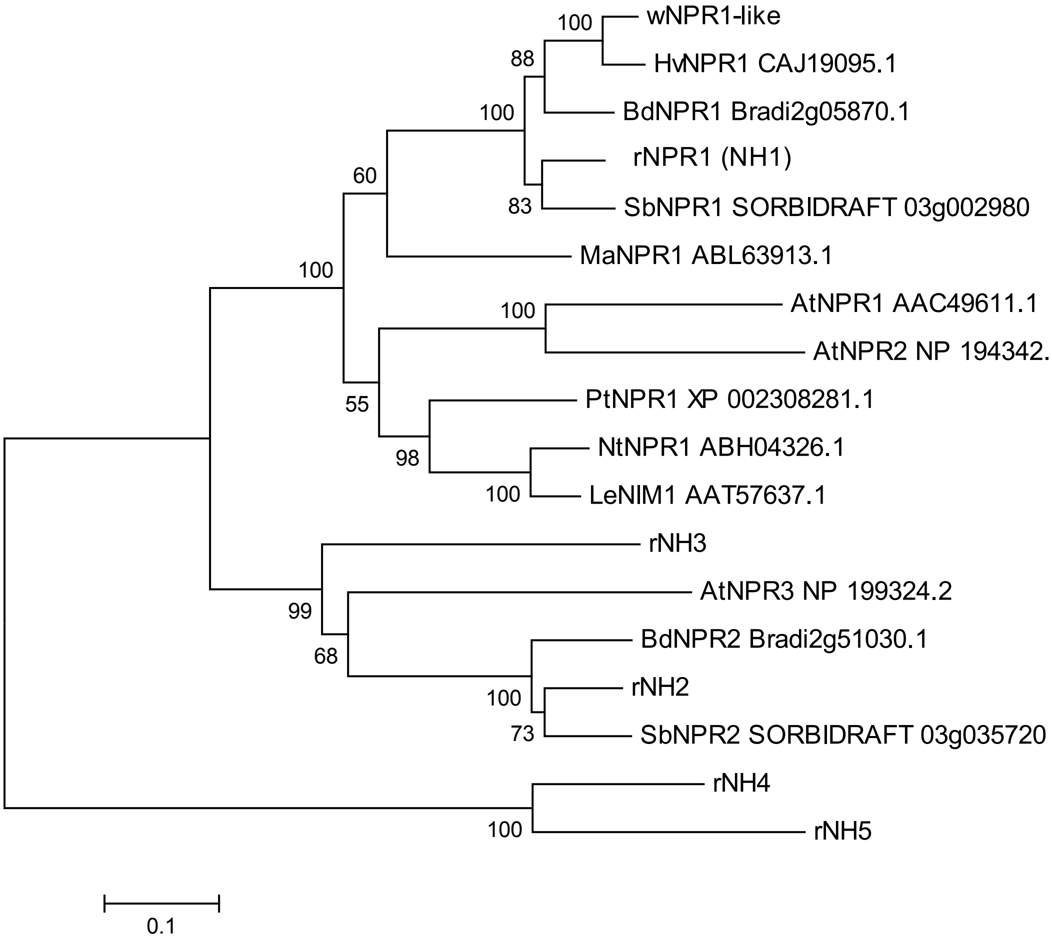

Figure S4 - Phylogeny of NPR1 homologs. The represented tree is the bootstrap consensus tree inferred from 1000 replicates generated using the Neighbor-Joining method [73]. The percentage of replicate trees in which the associated sequences clustered together in the bootstrap test (1000 replicates) are shown next to the branches. The analysis involved 18 amino acid sequences and 368 amino acid positions. All positions containing gaps and missing data were eliminated. Evolutionary analyses were conducted in MEGA5 [46].

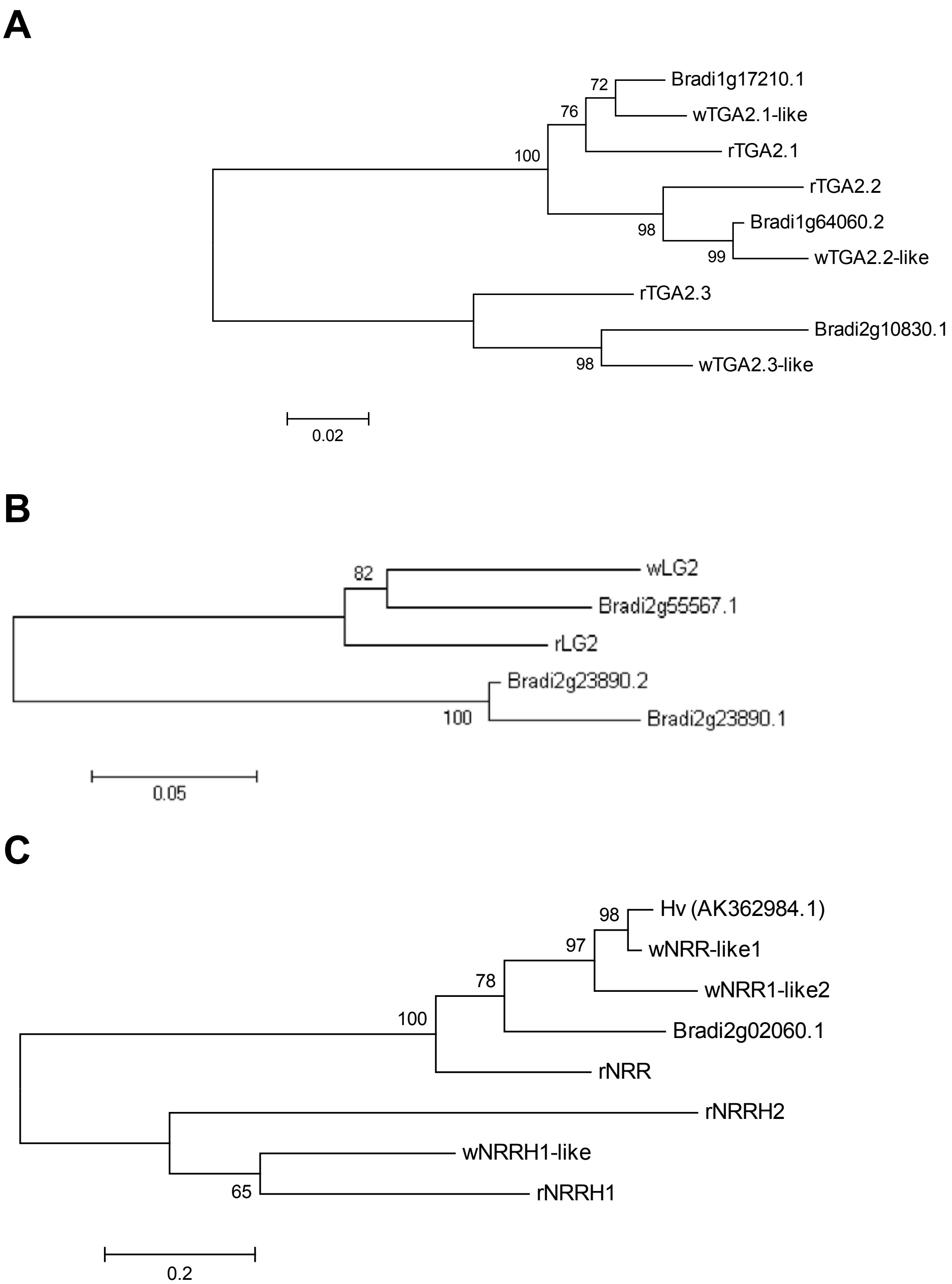

Figure S5 - Phylogeny of TGA (A), LG2 (B), and NRR (C) homologs. All represented trees are the bootstrap consensus trees inferred from 1000 replicates generated using the Neighbor-Joining method [73]. The percentage of replicate trees in which the associated sequences clustered together in the bootstrap test (1000 replicates) are shown next to the branches. All positions containing gaps and missing data were eliminated. Evolutionary analyses were conducted in MEGA5 [46].

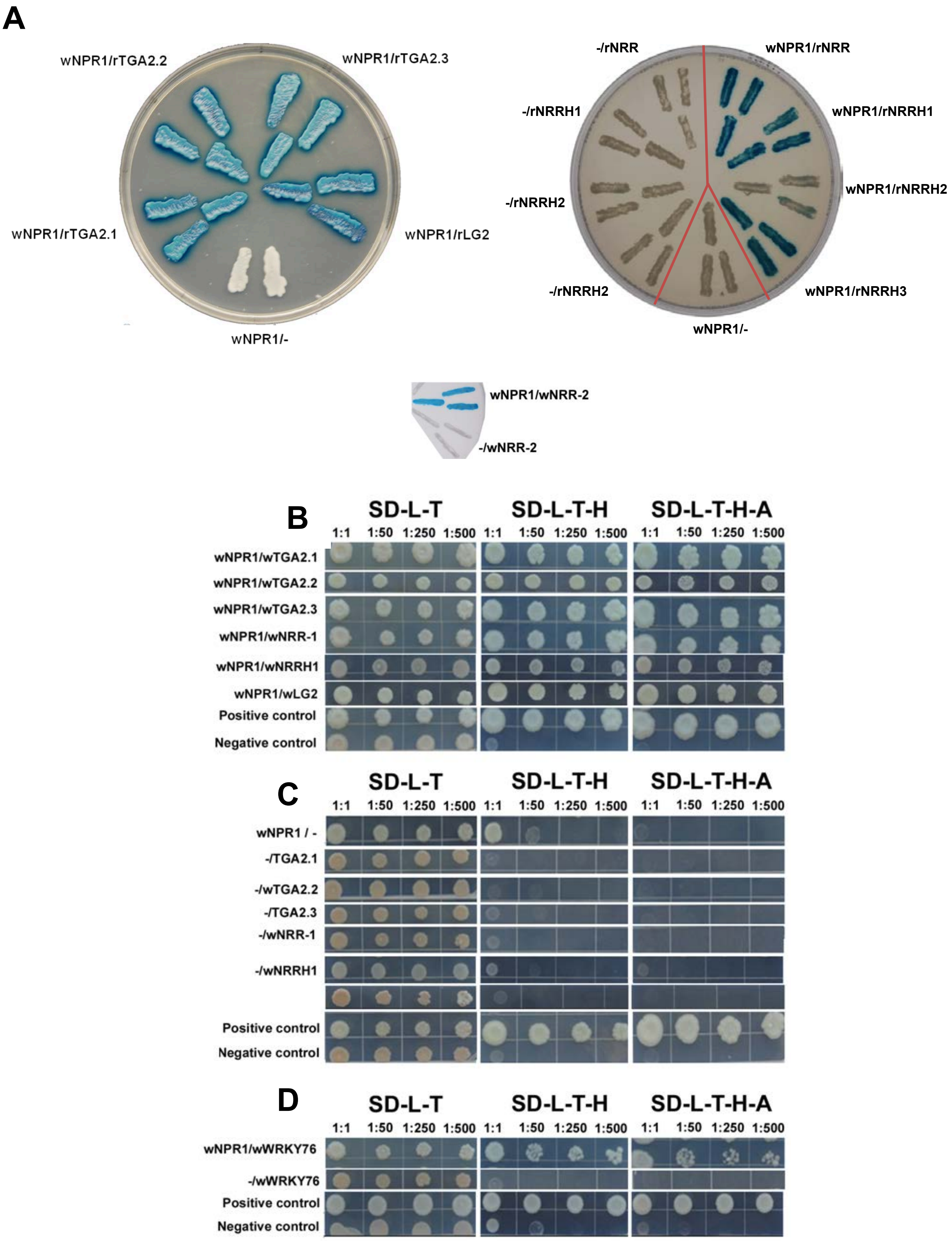
A
wNPR1/rTGA2.2
wNPR1/rTGA2.3
wNPR1/rTGA2.1
wNPR1/rLG2
wNPR1/-
-/rNRR
wNPR1/rNRR
-/rNRRH1
wNPR1/rNRRH1
-/rNRRH2
wNPR1/rNRRH2
-/rNRRH2
wNPR1/rNRRH3
wNPR1/-
wNPR1/wNRR-2
-/wNRR-2
B
SD-L-T
SD-L-T-H
SD-L-T-H-A
1:1 1:50 1:250 1:500
wNPR1/wTGA2.1
wNPR1/wTGA2.2
wNPR1/wTGA2.3
wNPR1/wNRR-1
wNPR1/wNRRH1
wNPR1/wLG2
Positive control
Negative control
C
SD-L-T
SD-L-T-H
SD-L-T-H-A
1:1 1:50 1:250 1:500
wNPR1 / -
-/TGA2.1
-/wTGA2.2
-/TGA2.3
-/wNRR-1
-/wNRRH1
Positive control
Negative control
D
SD-L-T
SD-L-T-H
SD-L-T-H-A
1:1 1:50 1:250 1:500
wNPR1/wWRKY76
-/wWRKY76
Positive control
Negative control

Figure S6 - Yeast-two-hybrid tests of interactions between the wheat orthologous copy of NPR1 (wNPR1-like) and known rice NPR1 interacting proteins (A), and between their orthologs in wheat (B & C). In (D): interaction tests between positive wXA21-like1 interacting wWRKY76 and wNPR1.

Autoactivation of clones was tested using empty bait (-/) or pray vectors (/-). In (A): strength of the interaction between proteins correlates with blue color intensity developed by yeast colonies. In (B & C): positive co-transformation in the Gal4 based system was tested in SD-L-T media, whereas positive interactions were tested in SD-L-T-H and SD-L-T-H-A median, in absence of histidine (-H) and both histidine and adenine (-H-A), respectively.

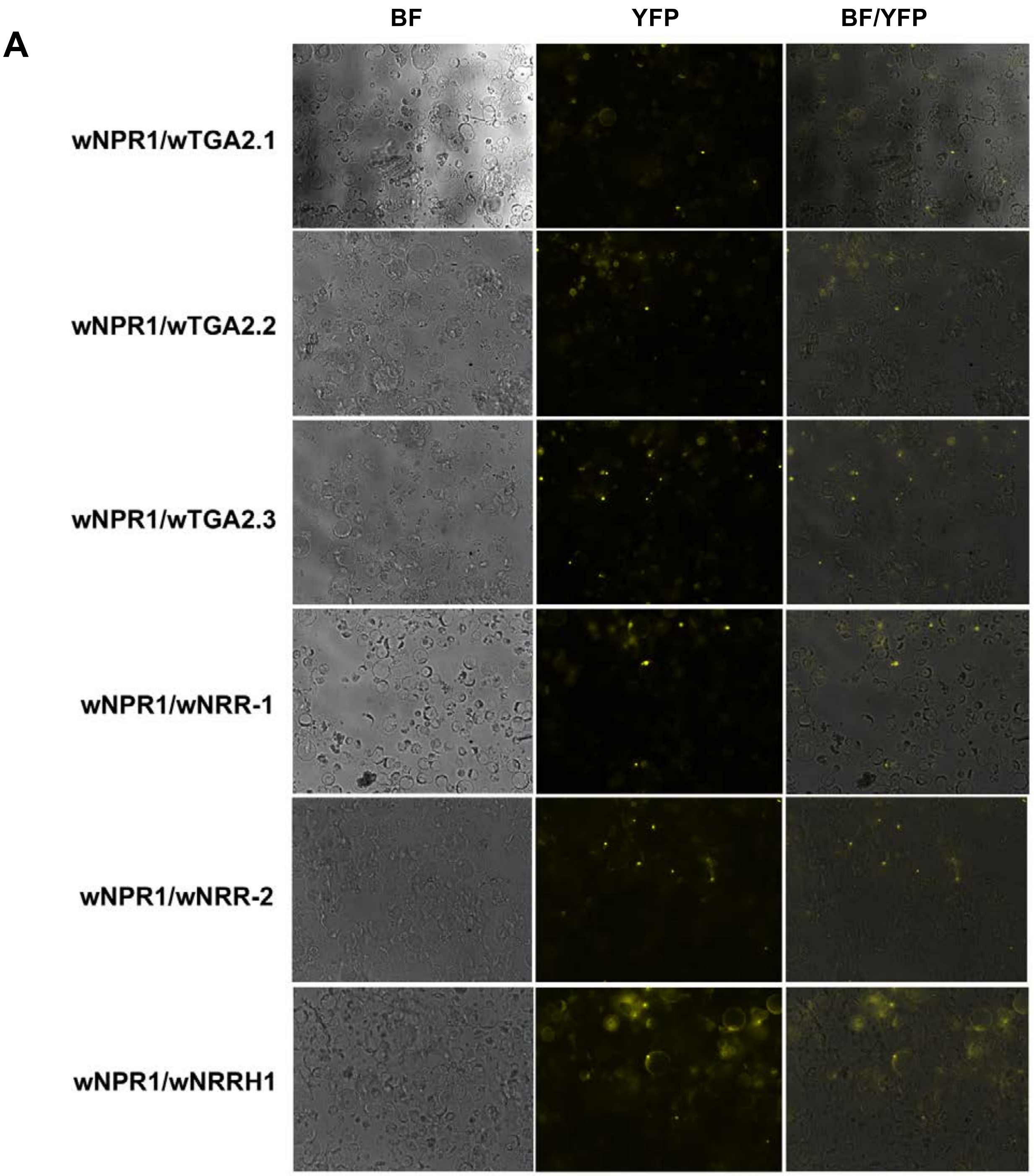
A
BF
YFP
BF/YFP
wNPR1/wTGA2.1
wNPR1/wTGA2.2
wNPR1/wTGA2.3
wNPR1/wNRR-1
wNPR1/wNRR-2
wNPR1/wNRRH1

B

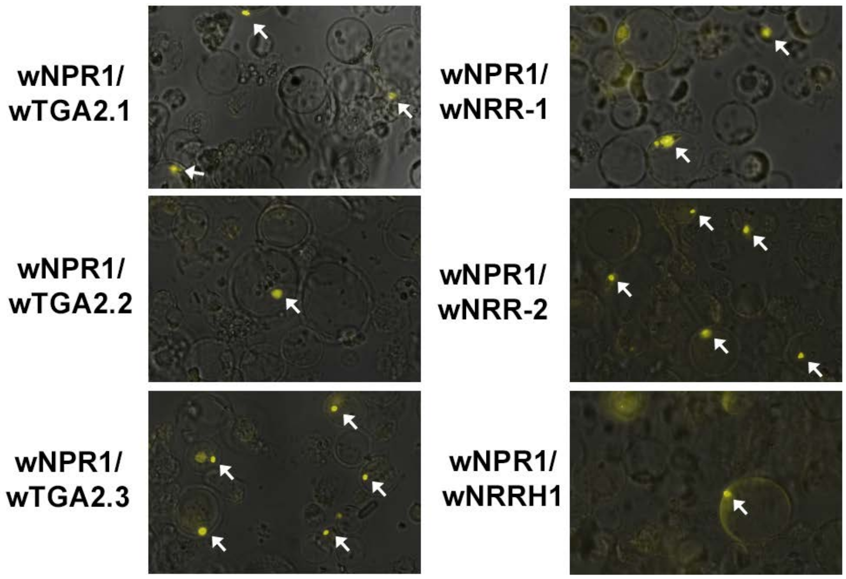
wNPR1/
wTGA2.1
wNPR1/
wNRR-1
wNPR1/
wTGA2.2
wNPR1/
wNRR-2
wNPR1/
wTGA2.3
wNPR1/
wNRRH1

C

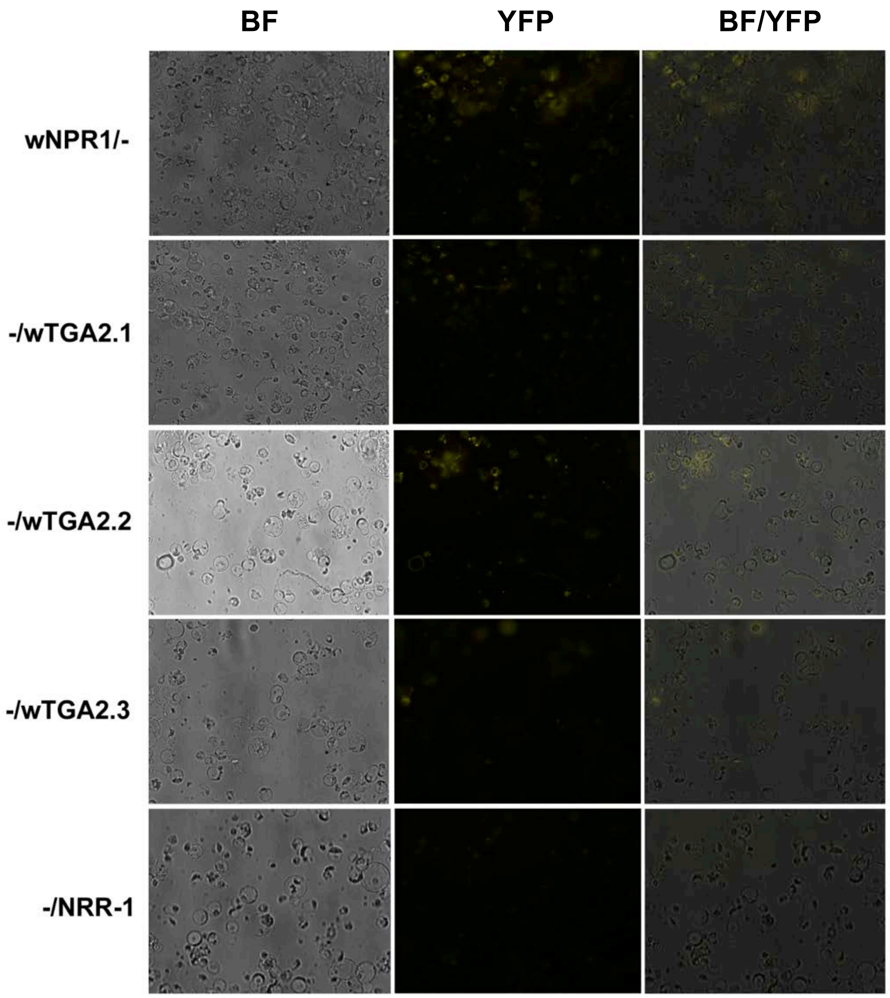
BF
YFP
BF/YFP
wNPR1/-
-/wTGA2.1
-/wTGA2.2
-/wTGA2.3
-/NRR-1

Figure S7 - BiFC assays showing positive interactions localized in the nuclei between wheat NPR1 and wheat TGAs and NRRs in rice protoplasts.

(A) Bimolecular fluoresence complementation assay (BiFC) was used to visualize protein-protein interactions in rice protoplasts. Images were taken using a Zeiss Axiovert 25 microscope with a 40× objective under either bright-field illumination (BF) or a YFP specific filter (YFP).
(B) Close-up images as in A (BF/YFP). Arrows point to nuclear localized YFP signals.

(C) Unfused YFPN-constructs were used as negative controls.

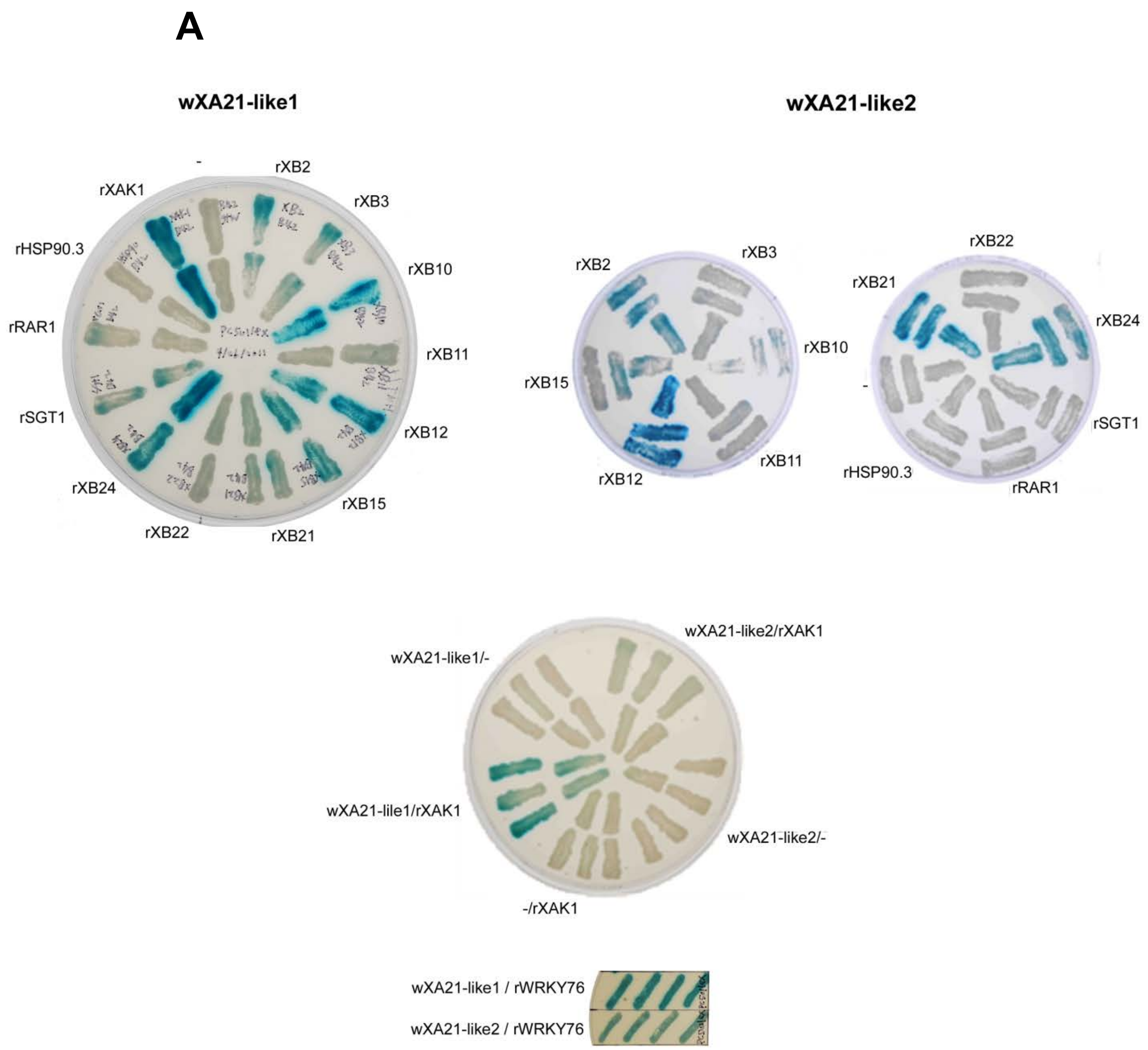

B

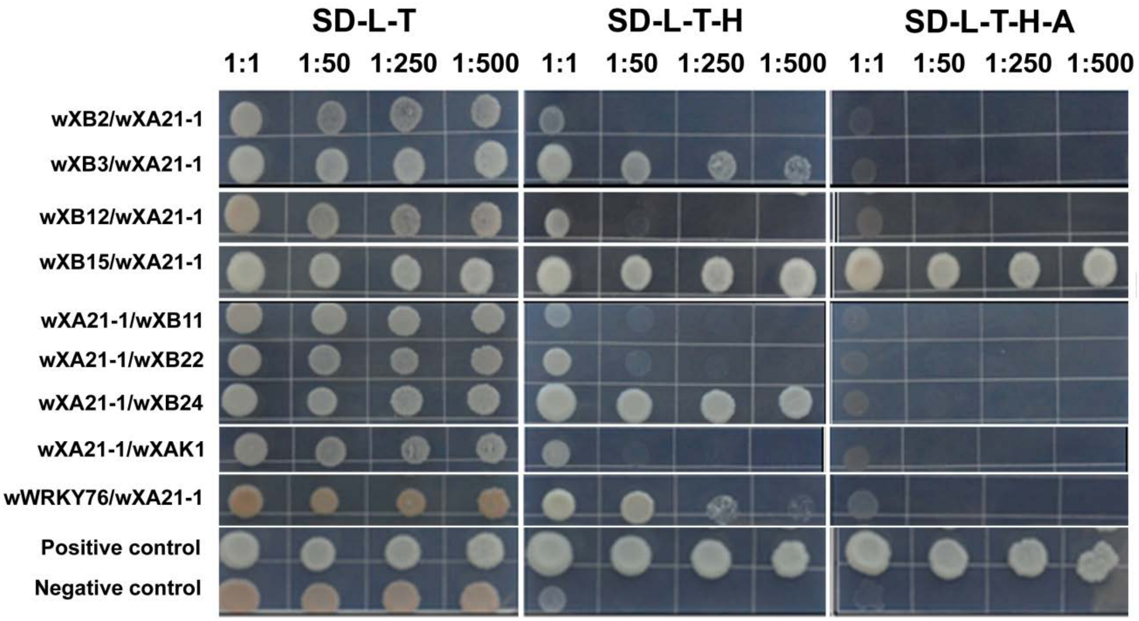
SD-L-T
SD-L-T-H
SD-L-T-H-A
1:1 1:50 1:250 1:500
wXB2/wXA21-1
wXB3/wXA21-1
wXB12/wXA21-1
wXB15/wXA21-1
wXA21-1/wXB11
wXA21-1/wXB22
wXA21-1/wXB24
wXA21-1/wXAK1
wWRKY76/wXA21-1
Positive control
Negative control

C

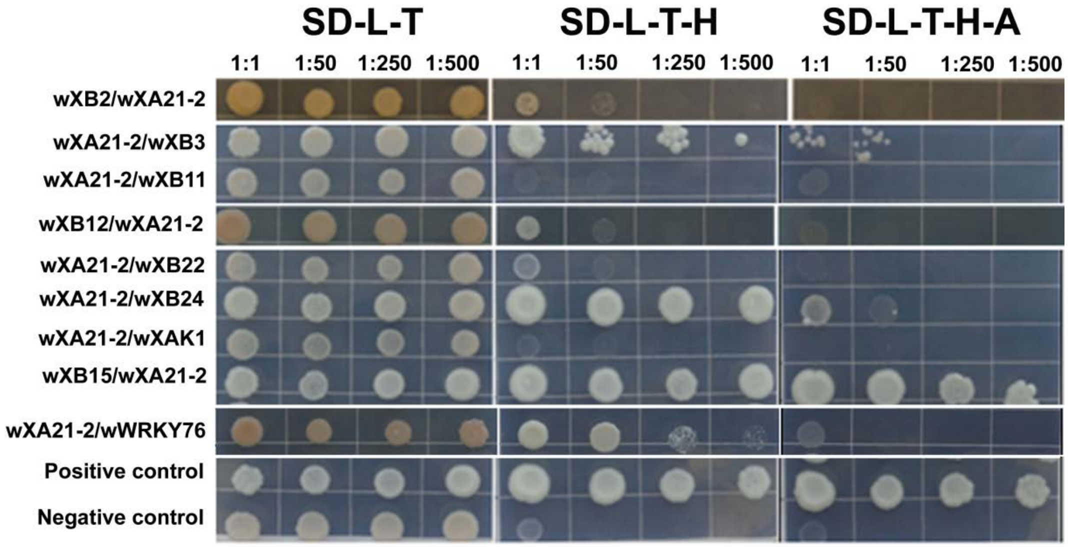
SD-L-T
SD-L-T-H
SD-L-T-H-A
1:1 1:50 1:250 1:500
wXB2/wXA21-2
wXA21-2/wXB3
wXA21-2/wXB11
wXB12/wXA21-2
wXA21-2/wXB22
wXA21-2/wXB24
wXA21-2/wXAK1
wXB15/wXA21-2
wXA21-2/wWRKY76
Positive control
Negative control

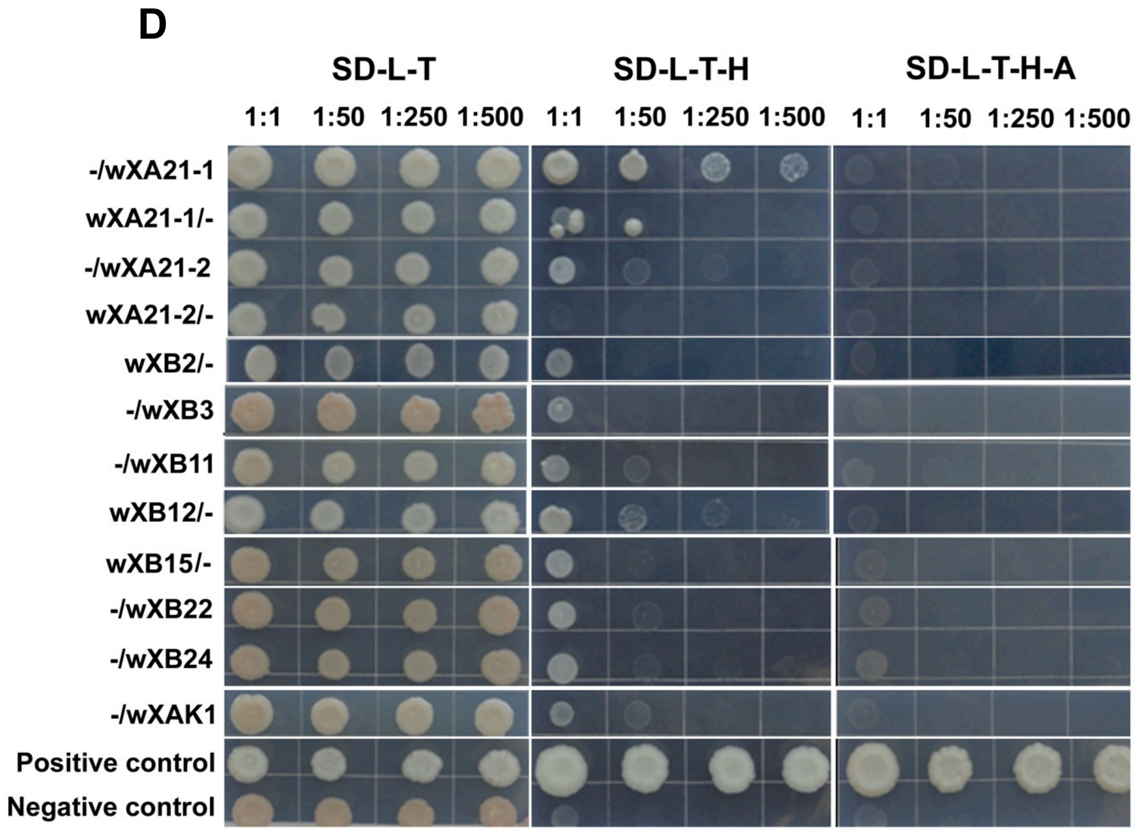

Figure S8 - Yeast-two-hybrid tests of interactions between the cytosolic domain of wheat XA21 copies (wXA21-like1 & wXA21-like2) and known XA21 interacting proteins (A) and their orthologous copies in wheat (B-D). Autoactivation of clones was tested using empty bait (-/) or pray vectors (/-). In (A & D): strength of the interaction between proteins correlates with blue color intensity developed by yeast colonies. In (B-D): positive co-transformation in the Gal4 based system was tested in SD-L-T media, whereas positive interactions were tested in SD-L-T-H and SD-L-T-H-A median, in absence of histidine (-H) and both histidine and adenine (-H-A), respectively. Interaction tests for all rXBs, with the exception of rXAK1 were carried out in [4].

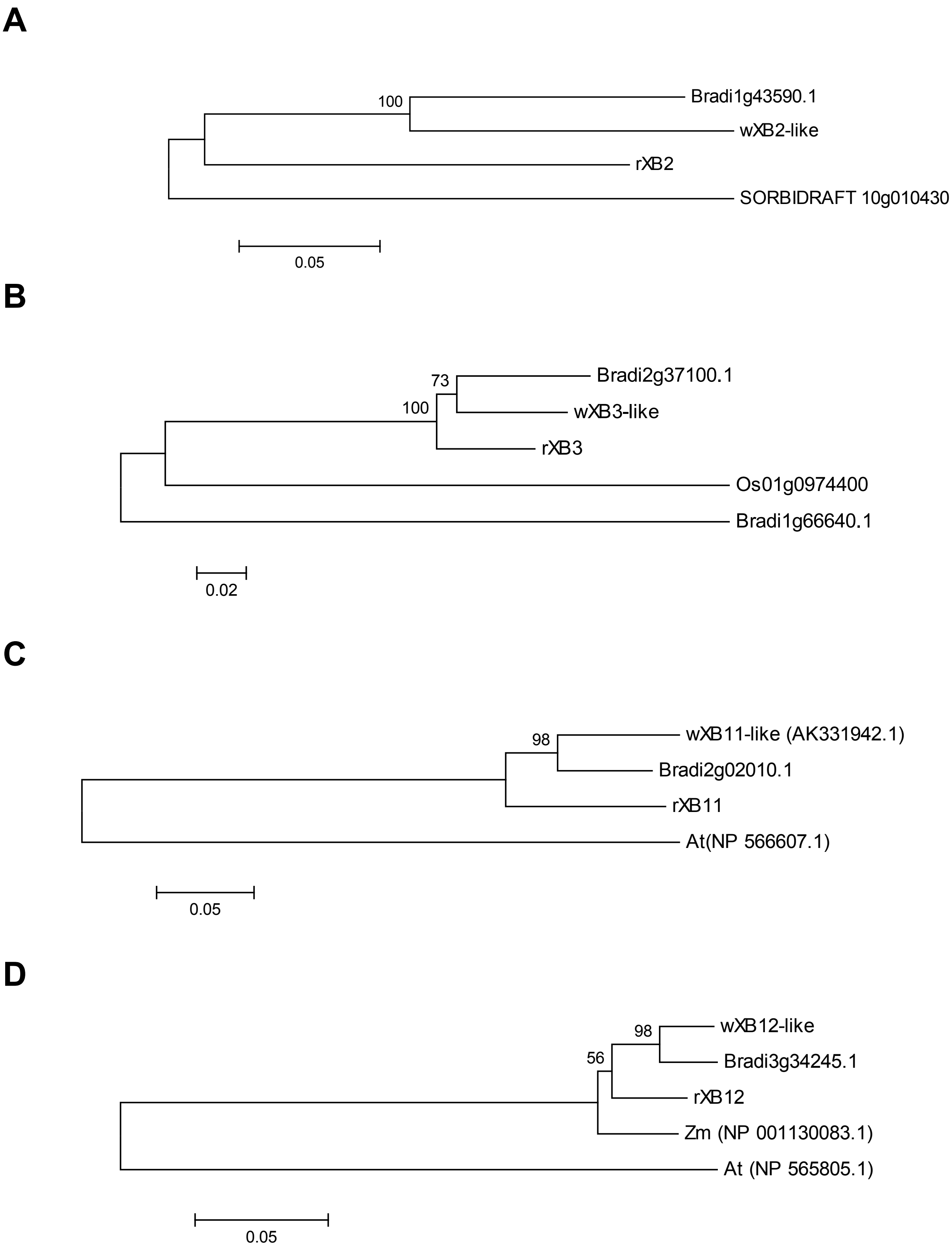
A
100
Bradi1g43590.1
wXB2-like
rXB2
SORBIDRAFT 10g010430
0.05
B
73
100
Bradi2g37100.1
wXB3-like
rXB3
Os01g0974400
Bradi1g66640.1
0.02
C
98
wXB11-like (AK331942.1)
Bradi2g02010.1
rXB11
At(NP 566607.1)
0.05
D
98
56
wXB12-like
Bradi3g34245.1
rXB12
Zm (NP 001130083.1)
At (NP 565805.1)
0.05

E

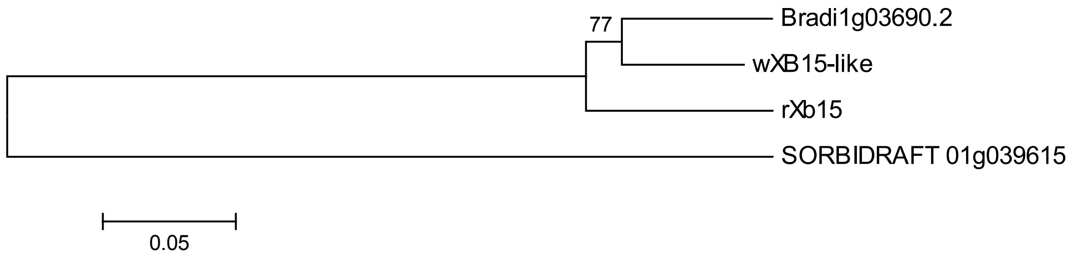
77
Bradi1g03690.2
wXB15-like
rXb15
SORBIDRAFT 01g039615
0.05

F

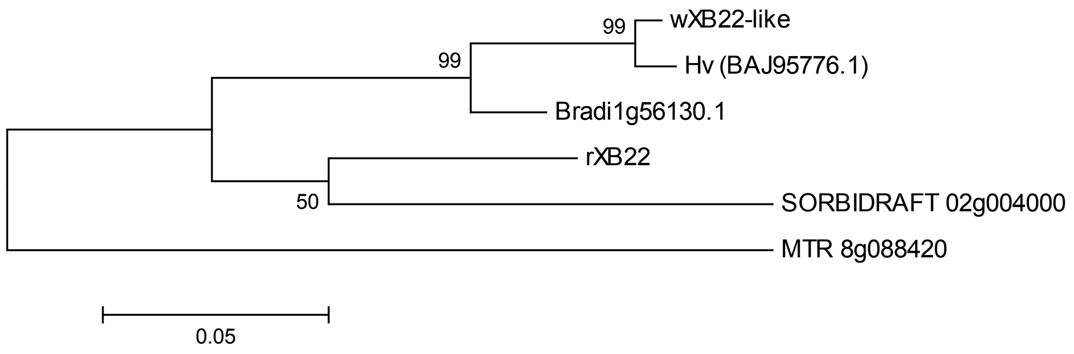
99
99
wXB22-like
Hv (BAJ95776.1)
Bradi1g56130.1
rXB22
50
SORBIDRAFT 02g004000
MTR 8g088420
0.05

G

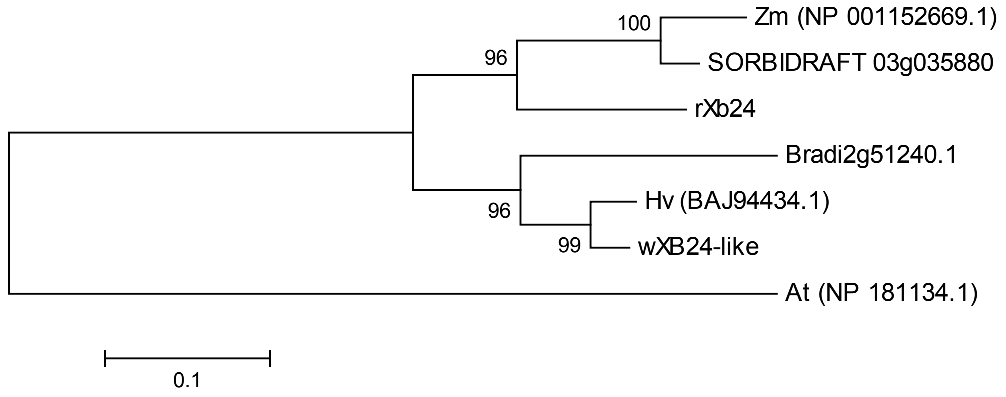
100
96
Zm (NP 001152669.1)
SORBIDRAFT 03g035880
rXb24
Bradi2g51240.1
96
Hv (BAJ94434.1)
99
wXB24-like
At (NP 181134.1)
0.1

H

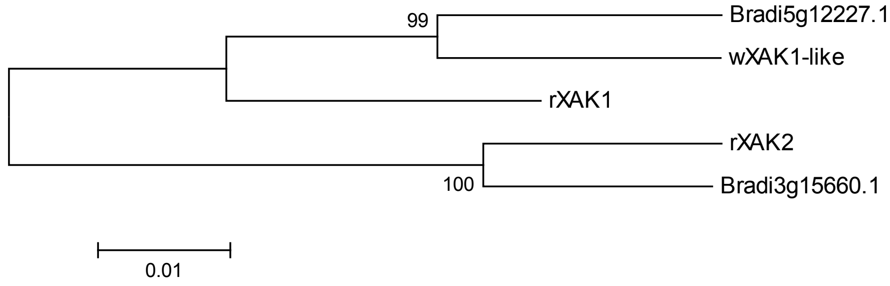

I

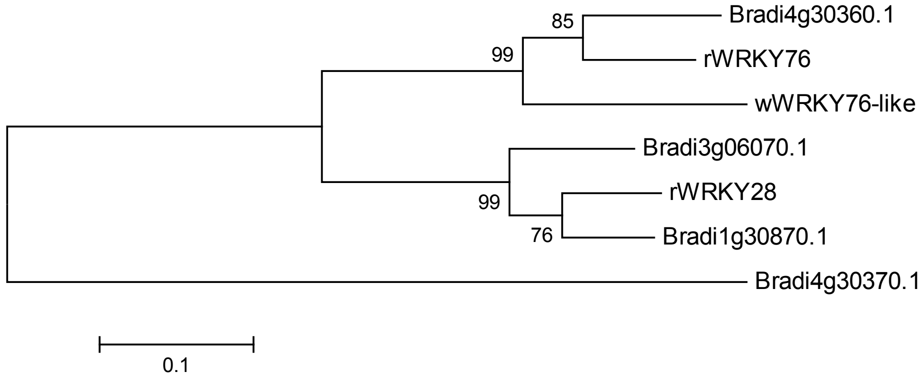

Figure S9 - Phylogeny of XA21 interacting proteins. (A) XB2; (B) XB3; (C) XB11; (D) XB12; (E) XB15; (F) XB22; (G) XB24; (H) XAK1; (I) WRKY76. All represented trees are the bootstrap consensus trees inferred from 1000 replicates generated using the Neighbor-Joining method [73]. The percentage of replicate trees in which the associated sequences clustered together in the bootstrap test (1000 replicates) are shown next to the branches. The analysis involved 25 amino acid sequences and 242 amino acid positions. All positions containing gaps and missing data were eliminated. Evolutionary analyses were conducted in MEGA5 [46].

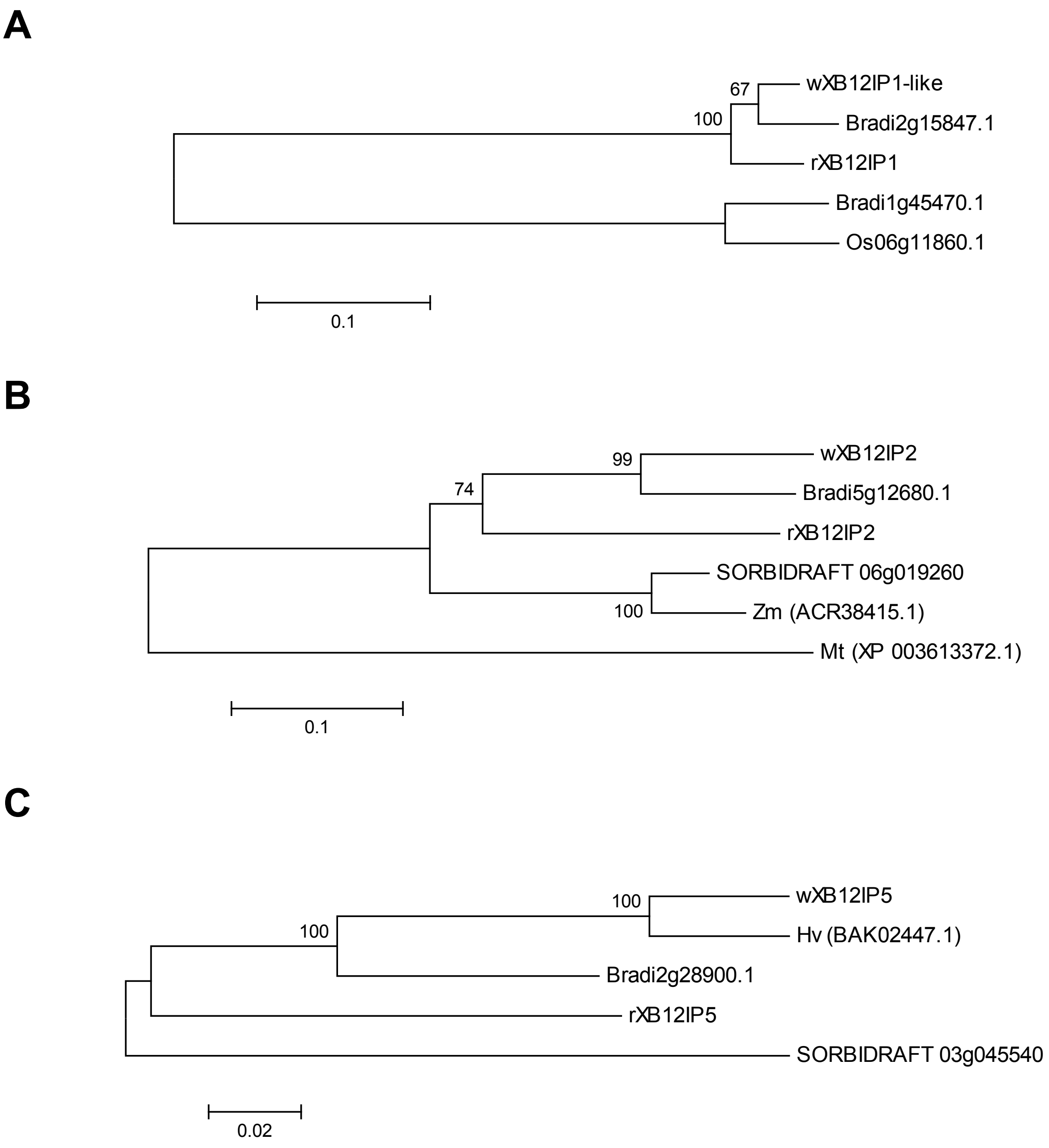

Figure S10 - Phylogeny of XB12 interacting proteins (wXB12IPs). (A) XB12IP1; (B) XB12IP2; (C) XB12IP5. All represented trees are the bootstrap consensus trees inferred from 1,000 replicates generated using the Neighbor-Joining method [73]. The percentage of replicate trees in which the associated sequences clustered together in the bootstrap test (1000 replicates) are shown next to the branches. All positions containing gaps and missing data were eliminated. Evolutionary analyses were conducted in MEGA5 [46].

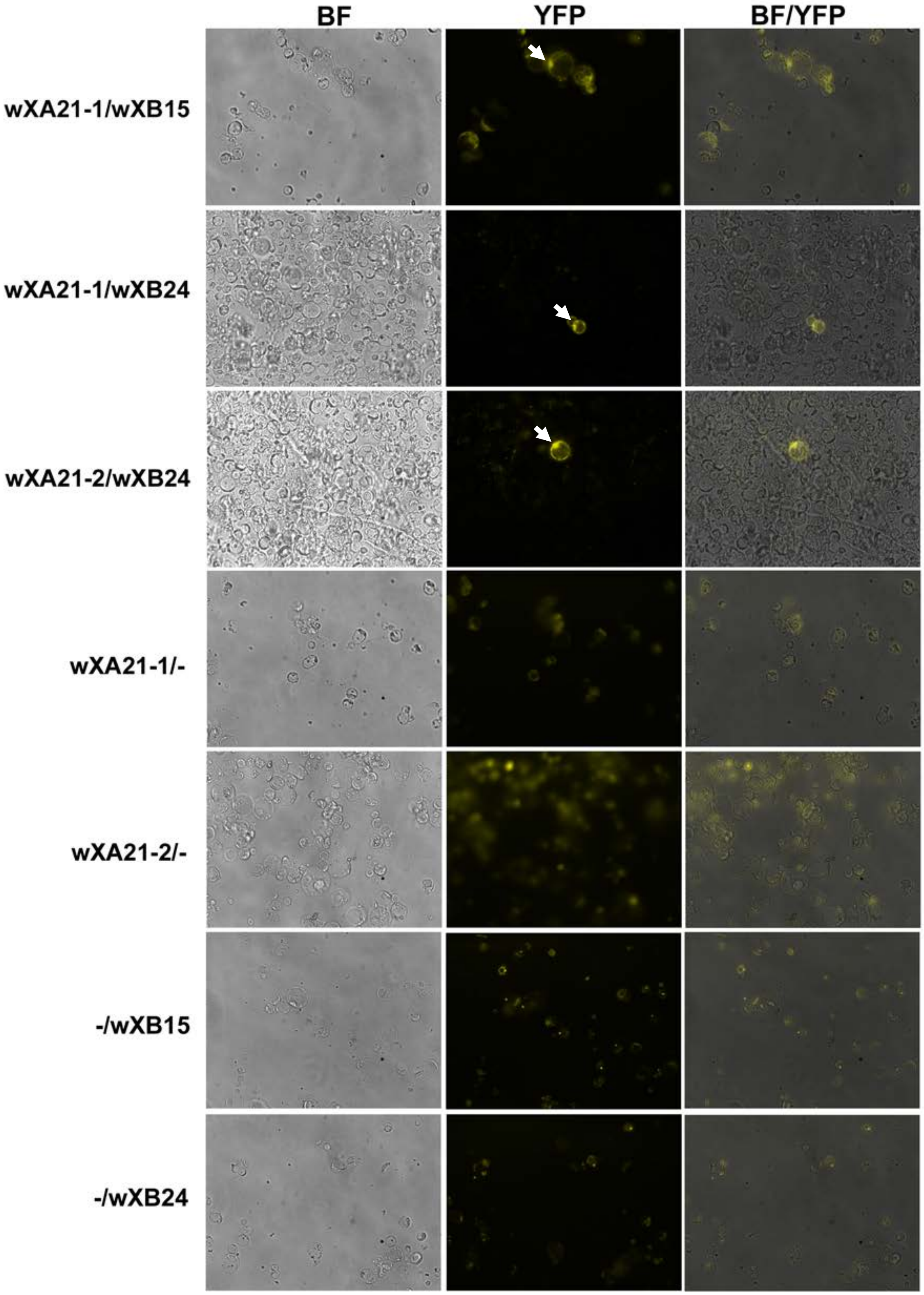
BF
YFP
BF/YFP
wXA21-1/wXB15
wXA21-1/wXB24
wXA21-2/wXB24
wXA21-1/-
wXA21-2/-
-/wXB15
-/wXB24

Figure S11 - BiFC assays showing positive interactions between wheat XA21-like proteins and wheat XBs. Bimolecular fluoresence complementation assay (BiFC) was used to visualize protein-protein interactions in rice protoplasts (arrowhead = positive YFP signal). Images were taken using a Zeiss Axiovert 25 microscope with a 40× objective under either bright-field illumination (BF) or a YFP specific filter (YFP). Unfused YFPN-constructs were used as negative controls.

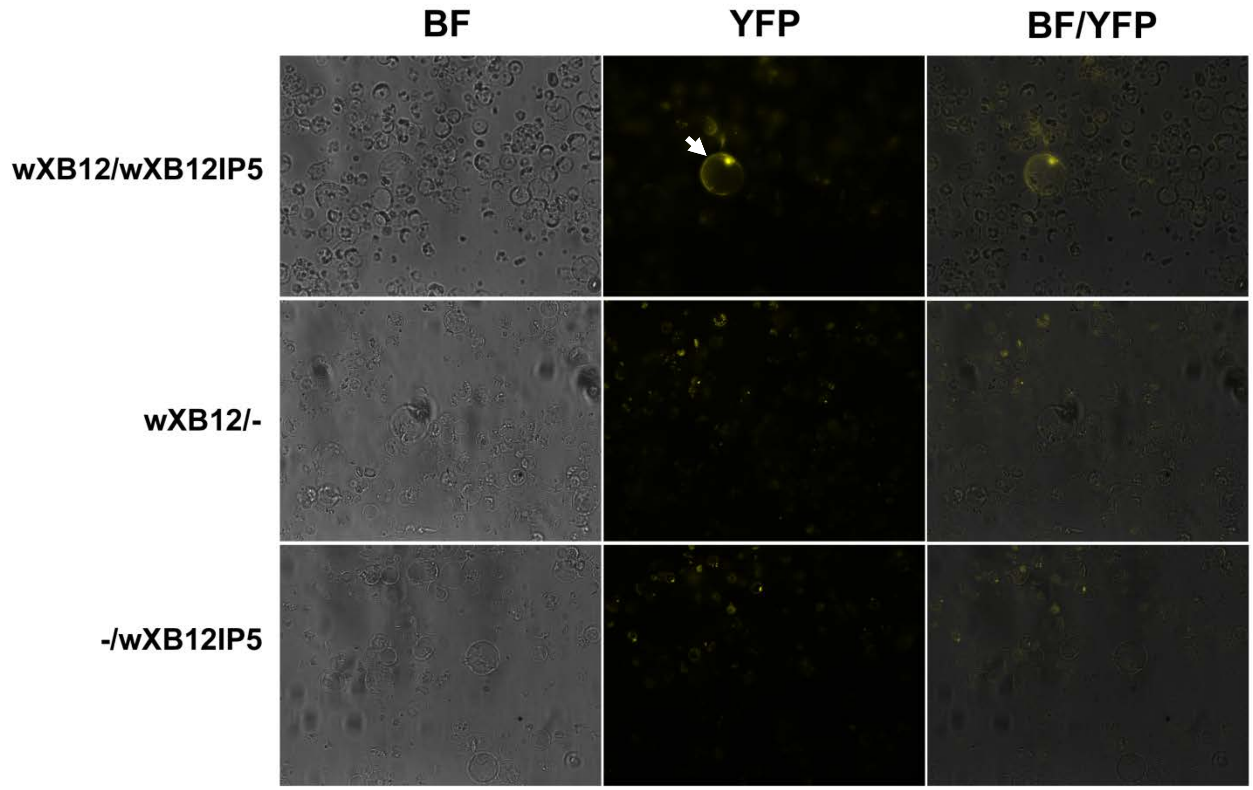

Figure S12 - BiFC assays showing positive interactions between wheat XB12 and wheat XB12IP5 in rice protoplasts. Bimolecular fluorescence complementation assay (BiFC) was used to visualize protein-protein interactions in rice protoplasts (arrowhead = positive YFP signal). Images were taken using a Zeiss Axiovert 25 microscope with a 40× objective under either bright-field illumination (BF) or a YFP specific filter (YFP). Unfused YFPN-constructs were used as negative controls.

Table S1 - Chromosome locations and putative synteny of the genes used in this study in rice, *B. distachyon* and wheat.

	Chromosome			
Gene	Rice	Brachy	Wheat[1]	Putative synteny[2]
XA21	11	4	5[3], 2[4]	Y[3],N[4]
XB2	6	1	7	Y
XB3	5	2	1	Y
XB11	1	2	3	Y
XB12	10	3	1	Y
XB15	3	1	5	Y
XB22	7	1	2	Y
XB24	1	2	3	Y
XAK1	4	5	2	Y
WRKY76	9	4	5	Y
XB12IP1	5	2	-	-
XB12IP2	4	5	2	Y
XB12IP5	5	2	1	Y
NPR1	1	2	3	Y
TGA2.1	7	1	2	Y
TGA2.2	3	1	4	Y
TGA2.3	1	2	3	Y
NRR	1	2	5[5], -5[6]	Y[5], -[6]
NRRH1	5	2	1	Y
LG2	1	2	3	Y
RAR1	2	3	2	N
HSP90.2	8	3	7	Y
HSP90.3	9	4	5	Y
SGT1	8	2	3	Y

[1] Locations on wheat chromosomes was determined by homology searches (BLASTN) against the wheat chromosome sequences available through the URGI bioinformatics platform (http://urgi.versailles.inra.fr).

[2] Synteny of homologous copies is hypothesized when genes resides on synthenic chromosomes in rice, *B. distachyon*, and wheat.

[3] wXA21-like1

[4] wXA21-like2

[5] wNRR-like1

[6] wNRR-like2
